# Soyasaponin and vertical microbial transmission: Maternal effect on the intestinal development and health of early chicks

**DOI:** 10.1002/imt2.70044

**Published:** 2025-05-20

**Authors:** Mingkun Gao, Shu Chen, Hao Fan, Peng Li, Aiqiao Liu, Dongli Li, Xiaomin Li, Yongfei Hu, Guofeng Han, Yuming Guo, Zengpeng Lv

**Affiliations:** ^1^ State Key Laboratory of Animal Nutrition and Feeding, College of Animal Science and Technology China Agricultural University Beijing China; ^2^ China Agricultural University‐Sichuan Advanced Agricultural & Industrial Institute Chengdu China; ^3^ Beijing Huadu Yukou Poultry Industry Co., Ltd. Beijing China; ^4^ Department of Medicine The University of Chicago Chicago Illinois USA; ^5^ Institute of Agricultural Facilities and Equipment Jiangsu Academy of Agricultural Sciences Nanjing China

**Keywords:** chick development, maternal nutrition, microbiota transmission, yolk sac, γ‐aminobutyric acid

## Abstract

Multiple factors, including genetics, nutrition, and health, influence the vertical transmission of microbiota from mothers to their offspring. Recent studies have shown that avian microbiota can be passed to the next generation via the eggshell and egg albumen. However, it remains unclear whether these microbial communities are regulated by nutrition and how they are associated with the host genotype. Chickens, with their controlled rearing conditions and stable genotypes, provide a promising model for investigating microbiome transmission in birds. This study aims to determine whether host genotype‐associated bacteria are vertically transmitted between generations, and how maternal nutritional intervention with soyasaponin modulates this microbial transfer, thereby shaping chick intestinal development and informing effective nutritional strategies. We established a microbial vertical transmission model across various anatomical sites in breeder hens, chicken embryos, and chicks. Avian gut microbiota and reproductive tract microbiota can both be found in chicks at various developmental stages. Supplementing breeder hen diets with soyasaponin interacts with vertically transmitted *Bifidobacterium adolescentis* to produce γ‐aminobutyric acid. This compound modulates offspring intestinal development through distinct mechanisms in chick epithelial cells, including the inhibition of LC3 and caspase3‐associated autophagy and apoptosis pathways, as well as the promotion of proliferation and differentiation pathways mediated by LGR5 and Olfm4. Our study highlights that avian gut and reproductive tract microbiota are transmitted to chicks through the cloaca, with the yolk sac also being instrumental in this vertical transfer. The incorporation of soyasaponin in avian diets affects microbial transfer, providing a theoretical basis for studying maternal effects in poultry and formulating corresponding dietary strategies.

## Highlights

- The yolk sac plays a crucial role in the vertical transmission of maternal microbiota in birds.
- Soyasaponin (SS) facilitates the colonization of *Bifidobacterium* in both maternal and offspring microbiota, contributing to microbial inheritance.
- SS interacts with *Bifidobacterium adolescentis* (*B. adolescentis*), regulating embryonic development through microbial and metabolic pathways.
- SS enhances the production of Gamma‐aminobutyric acid (GABA) by *B. adolescentis*, thereby shaping chick gut development through mechanisms influencing proliferation, differentiation, and apoptosis.

## Graphical abstract

This study investigates the vertical transmission of maternal microbiota in chickens and how it is modulated by maternal soyasaponin supplementation. Using a model spanning the reproductive tract, egg components, and developing chicks, we show that maternal gut and reproductive microbiota, including *Bifidobacterium adolescentis* (*B. adolescentis*), are transmitted via the yolk sac. Soyasaponin enhances this transmission and promotes *B. adolescentis*‐derived Gamma‐aminobutyric acid (GABA) production. GABA modulates chick intestinal development by suppressing autophagy and apoptosis pathways and activating proliferation and differentiation. These effects are mediated through GABA receptors. The study highlights the interplay between host genetics, maternal diet, and microbiota inheritance, offering insights into nutritional strategies to optimize offspring intestinal health.

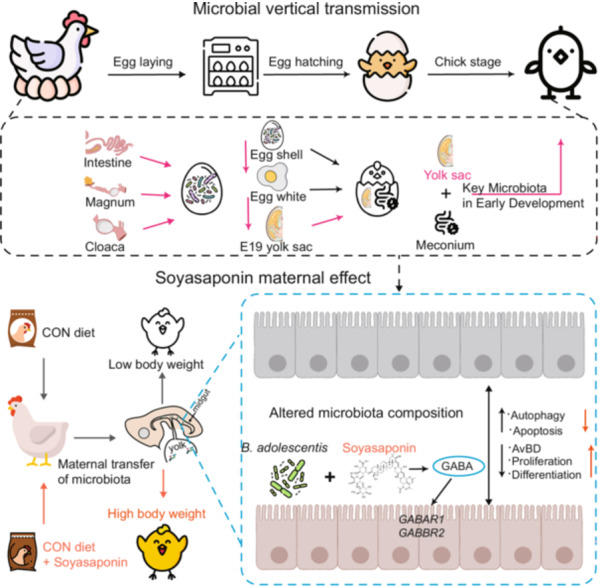

## INTRODUCTION

Maternal effect is a critical biological phenomenon observed across the animal kingdom. It involves the direct influence of the maternal on the external characteristics, physiological traits, and reproductive performance of their offspring [1]. Initially, there was a hypothesis that maternal effects in mammals could be mediated through various mechanisms, including epigenetic modifications, nutritional factors, immune factors, hormonal signaling, and transmission of the microbiome [2]. The early establishment of microbial communities during infancy is a critical process that profoundly influences the physiological, and long‐term health outcomes of mammals [3]. When investigating the dynamics of maternal‐infant microbiota, various factors, including the mode of childbirth, breastfeeding, and environmental conditions, play pivotal roles in shaping the composition and structure of the neonatal gut microbiome [4], which in turn substantially impact the physiological well‐being of newborns, such as the development, and overall health [5]. This implies that maternal‐source microbial communities may have significant implications for maternal effects.

Efficient vertical transfer is also crucial for gut microbes, especially when considering the benefits of early niche colonization [6]. Microbial communities with transmissibility have a more significant contribution to the host genotype [7]. In contrast to mammals, avian embryos without direct anatomical connection with the maternal body, lacking direct connections to the maternal body, such as an umbilical cord, placenta, or amniotic fluid [8]. The primary route of microorganism transmission in chickens is through the oviduct, wherein microorganisms are encapsulated in the egg whites and conveyed to the embryos, ultimately leading to colonization [9]. After detachment from the maternal source, microbial communities in the egg white, yolk sacs, and embryonic intestines within fertilized eggs undergo temporal and spatial change [10]. In particular, after hatching, the residual yolk within the body serves as a nutritional source for newborn chicks. This process plays a critical role in the early development of the intestinal microbiota in chicken embryos. Research indicates that there are already microorganisms in the early yolk sac, and their abundance is significantly higher than in the egg white [11]. This finding implies that the microorganisms provided by the yolk sac may be one of the critical factors promoting the early development of chick microbiota.

Mothers with varying dietary formulations and physical characteristics may exert distinct influences on offspring gut microbiota and intestinal development through the vertical transmission of microbes and microbe‐promoting factors [12]. Wild birds and poultry primarily consume a diet of leguminous plants and grains, which are rich in flavonoid compounds. Recent research indicates that genistein, present in the egg whites and yolks of the maternal diet, significantly influences the colonization of microbial communities in the offspring [13]. Soyasaponin (SS) is a naturally occurring pentacyclic triterpenoid found abundantly in soybeans. Our previous studies have demonstrated that in the presence of intestinal microbiota, Soyasaponin facilitates B lymphocyte maturation and bolstering humoral immune function in birds [14]. The interaction between soyasaponin and the gut microbiota produces metabolites that promote gastrointestinal health. This observation holds significance as it may lead to a better establishment of intestinal immunity in the early chick stage, ultimately improving intestinal development and body growth. While the existence of adaptive maternal effects is evident, the mechanisms of transfer and their specific benefits to offspring remain unclear. In chicken embryos, initial nutrient allocation prioritizes muscle and skeletal development over intestinal growth [15], emphasizing the importance of nutritional supplementation during the embryonic window. Current research corroborates that stimulating intestinal development before hatching is feasible, mainly through in ovo injection. Nevertheless, implementing this method in practical production settings poses considerable challenges. Maternal nutritional strategies can promote early embryonic intestinal development [16]. Increasing evidence suggests that heritable bacteria are integral components of the gut microbiota interaction network [17]. Manipulating these transmissible bacteria in chickens may therefore represent an effective strategy for optimizing maternal nutrition. However, the mechanisms underlying these effects remain unclear due to various transmission methods, including nutrient deposition, vertical microorganism transmission, and metabolic products from nutrient–microbe interactions.

This study aims to elucidate the role of maternal microbial transfer in maternal effects and to identify the sources responsible for driving the early maturation of chick microbiota. We carried out an in‐depth analysis of the entire egg formation process within the maternal intestinal and reproductive systems, particularly emphasizing the unique physiological structures in birds and their relationship with microbial transmission. In addition, our primary focus was investigating the mechanisms by which dietary soyasaponin interacts with microorganisms in the maternal effect strategy.

## RESULTS

### Yolk sac as a potential conduit for maternal microbiota vertical transmission

**FIGURE 1:**
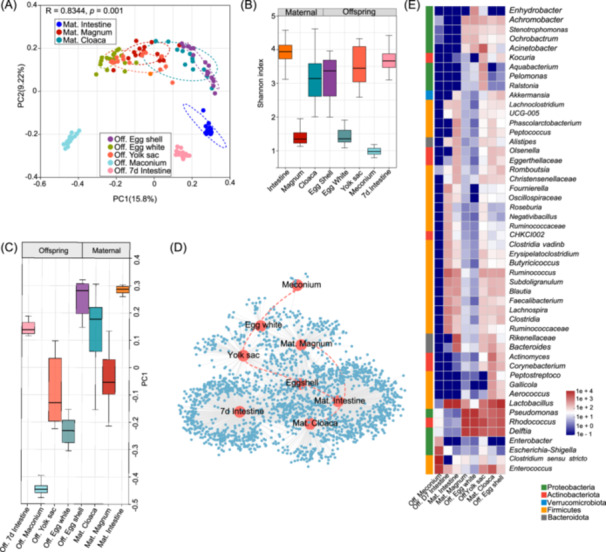
Colonization and body site specificity of the maternal and offspring. (A) Principal coordinate analysis (PCoA) of the unweighted UniFrac distances for maternal cecum (*n* = 16), magnum (*n* = 15) and cloaca (*n* = 16) samples, and offspring egg shell (*n* = 16), egg white (*n* = 15), E19 yolk sac (*n* = 15), meconium (*n* = 16) and 7 d cecum (*n* = 16) samples. Ellipses represent a 95% CI. (B) The Shannon index of each site. (C) Values of PCoA axis one for each site. (D) Bipartite networks demonstrate the shared microbiota across different locations. (E) The relative abundance of bacterial genera at different body sites.

**FIGURE 2:**
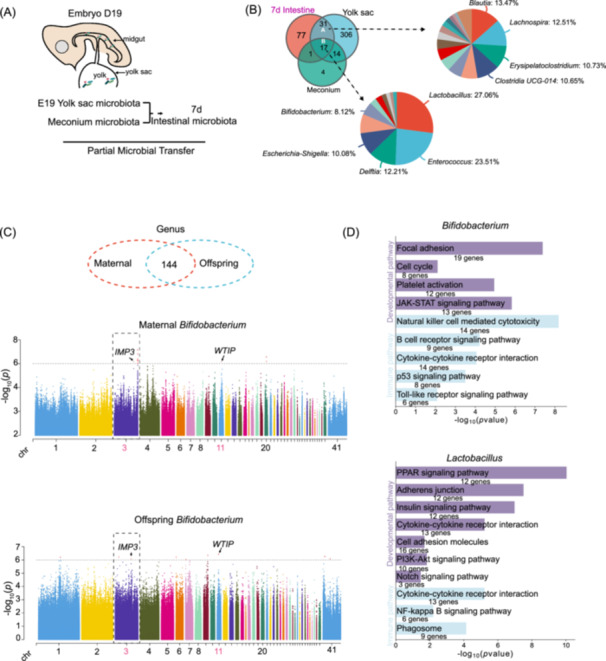
Host genotype regulates the vertical transmission of maternal microbiota. (A) During the embryonic stage in chickens, the yolk sac facilitates both nutrient absorption and the transmission of microbiota to the gut, a process observable up to 7 d post‐hatch. (B) Conduct Venn diagram analysis on meconium, E19 yolk sac, and 7 d chick's intestinal. The labeled intersections represent bacterial genera that can be transmitted individually. Corresponding pie charts illustrate the types and proportions of these bacterial genera. (C) The heritability and significant variants of the phenotype in maternal and offspring. Manhattan plot of *Bifidobacterium*. The significance threshold was 1/n single nucleotide polymorphism (SNP) = 1e‐06. (D) Kyoto Encyclopedia of Genes and Genomes (KEGG) functional pathways of heritable variants associated with *Bifidobacterium* and *Lactobacillus* in offspring.

To assess the potential influence of maternal nutrition on the microbiota of newly hatched offspring, we analyzed the community composition and structural characteristics of both maternal and neonatal microbiota. Notably, there were discernible clusters for maternal intestinal, oviduct, and cloaca microbiota, as well as clusters for egg white, yolk sac, and meconium microbiota (Figure 1A–C). Network analysis highlighted a strong link between the eggshell microbiota and the magnum and cloaca, implying a microbial circulation within the reproductive tract (Figure 1D). The microbial structure in these embryonic sites differed from the maternal's microbiota, suggesting site‐specific microbial compositions (Figure 1E). These findings suggest that fetal compartments with maternal microbial similarities are likely primary channels for vertical microbial transmission. Source tracker analysis unveiled that a significant portion of the eggshell microbiota originates from the oviduct and cloaca, and a considerable part from the maternal intestinal microbiota (Figure S1A). In the yolk sac, we identified core bacterial genera *Lactobacillus*, *Clostridium*, and *Enterococcus*, which can be transmitted through the cloaca and eggshell (Figure S1B). In genus‐level Venn analysis of the yolk sac and 7 d intestinal microbiota, we identified 31 shared genera, prominently featuring *Clostridia UCG‐014*, *Blautia*, and *Lachnospira* (Figure 2A,B). This highlights the significant role of the yolk sac microbiota in early chick gut development. Correlation analysis demonstrated that the microbiota structure of the yolk sac shares greater similarity with that of the maternal intestine, magnum, and cloaca (Figures S1C, S2). Further analysis among the meconium, yolk sac contents, and gut microbiota of 7‐day‐old chicks revealed 17 shared genera, with *Lactobacillus*, *Enterococcus*, *Escherichia*‐*Shigella*, and *Bifidobacterium* being the most common (Figure 2B). These findings suggest that these genera are capable of vertical transmission, colonizing both the yolk sac and the gut during the embryonic stage.

### Maternal vertical transmission of microbes is genotype‐dependent

To further demonstrate the potential transmission of microbiota between the maternal and offspring generations, we performed a genome‐wide association study (GWAS) analysis on host genetic variations in both parents and offspring of the transmitted microbiota. In the transmission of microbiota between the mother and offspring, a total of 277 and 59 significant associations were identified in the maternal and offspring microbiota, respectively (Figure 2C, Table S1). Additionally, eight SNP variants were identified in the maternal microbiota and nine in the offspring microbiota that were influenced by *Bifidobacterium* (−log_10_ (*p*) > 6). The results revealed that the transmitted microbiota showed the same heritable variations in both the maternal and offspring (Figure 2C). Notably, significant variations at chr3:109596245, chr3:109625955, chr3:109393549, chr3:109654405, chr3:109466958, chr3:109437289, chr3:109660517, chr3:109402591, and chr3:109725716 were found in *Bifidobacterium* (Tables S1, S2). These variations were annotated to the genes *CD2AP*, *ENSGALT00015045722*, Phospholipase A2 Group VII (*PLA2G7*), *ENSGALT00015045729*, Insulin‐like Growth Factor 2 mRNA‐Binding Protein 3 (*IMP3*), Meprin A Subunit Alpha (*MEP1A*), Opsin 5 (*OPN5*), and Patched Domain Containing 4 (*PTCHD4*) (Tables S2, S3). Similarly, *Lactobacillus*, *Bacteroides*, *Enterococcus*, and *Escherichia*‐*Shigella* were found to share common chromosomal regions between the mother and offspring, with significant colocalized variations identified (Figure S3).

In the results, the core genera *Bifidobacterium* and *Lactobacillus* in offspring are regulated by multiple heritable variants, which are closely associated with immune function and development (Tables S4, S5). The genes regulating *Bifidobacterium* are enriched in KEGG pathways such as Natural Killer Cell‐Mediated Cytotoxicity, B Cell Receptor Signaling Pathway, and p53 Signaling Pathway. Meanwhile, the genes regulating *Lactobacillus* are enriched in pathways including the PPAR Signaling Pathway, Insulin Signaling Pathway, and PI3K‐Akt Signaling Pathway (Figure 2D).

### Altered maternal microbiota composition across distinct maternal microenvironments in soyasaponin

**FIGURE 3:**
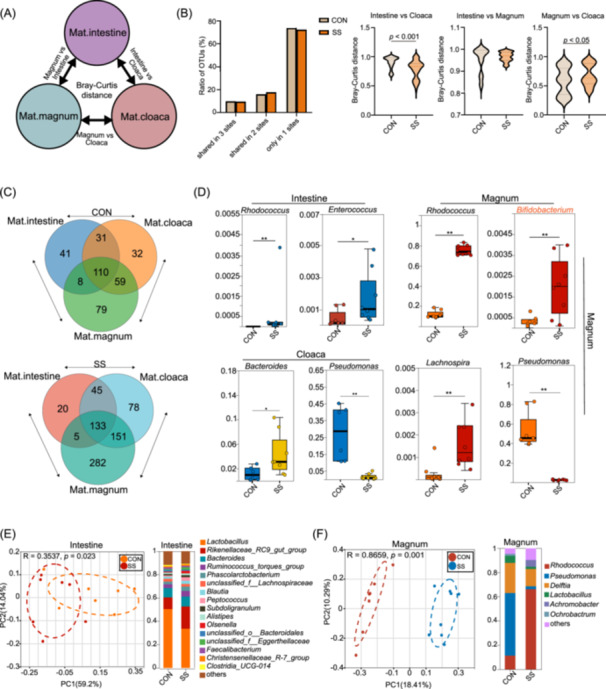
Microbial variations of the breeder broilers supplemented with soyasaponin. (A) Microbiota are transmitted within the breeder chicken through the intestine, magnum, and cloaca. (B) Proportions of shared operational taxonomic units (OTUs) among maternal intestinal, magnum, and cloaca microbiota. Shared OTU denotes that a certain OTU was detectable in two or three body sites. (C) Venn diagram of soyasaponin (SS) and CON groups at the genus level. (D) Significant differences in the shared bacterial genera among the three maternal sites. (E, F) Beta diversity and relative abundance at the genus level in the intestine and magnum of breeder chickens.

After clarifying the patterns of vertical microbial transmission between maternal and offspring, the next step is to further investigate the regulatory role of soyasaponin (SS) nutritional interventions in this process. Analysis of microbial changes in breeder hens receiving SS supplementation revealed a higher number of shared operational taxonomic unit in all three body sites compared to the control group (Figure 3A,B). Specifically, shared genera between the cloaca and magnum in the SS group were three times higher than those in the control group (Figure 3C). Wilcoxon rank‐sum tests on intestinal, magnum, and cloaca samples identified significant differences in 5, 8, and 10 genera, respectively (Figure S4A). Among these, *Rhodococcus* exhibited consistent changes in both magnum and intestinal samples (Figure 3D). Following SS treatment, beneficial genera for fetal development, including *Pseudomonas*, *Bifidobacterium*, and *Bacteroides*, showed a significant increase in abundance (Figure 3D). Principal coordinates analysis (PCoA) demonstrated distinct microbial structures in the intestine, magnum, and cloaca samples between the control and SS treatment groups (Figures 3E,F, S4B).

Dietary SS significantly improved the reproductive tract microbial structure and enhanced the reproductive performance of broiler breeders. Within 7 weeks of SS treatment, the average egg production rate significantly increased (Figure S5A). Furthermore, levels of estrogen (E2), follicle‐stimulating hormone (FSH), and luteinizing hormone (LH) also exhibited significant increases (Figure S5B). Dietary SS promoted intestinal development in broiler breeders, resulting in a significant increase in villus height (VH) and villus crypt ratio (VCR), coupled with a significant reduction in crypt depth (CD) (Figure S5C). Dietary SS significantly decreased the serum levels of the cytokine Interleukin‐2 (IL‐2) (Figure S5D). Additionally, dietary SS had a positive impact on the health of the oviduct, as it significantly reduced the mRNA expression of the cytokine IL‐2, and notably improved the percentage of healthy chicks (Figure S5E,F). The egg production rate is positively correlated with *Bacteroides*, *Bifidobacterium* while E2, FSH, and LH are positively correlated with *Clostridium sensu stricto, Bifidobacterium,* and *Rhodococcus* (Figure S5G).

### Reshaped microbiota in offspring following maternal soyasaponin supplementation

**FIGURE 4:**
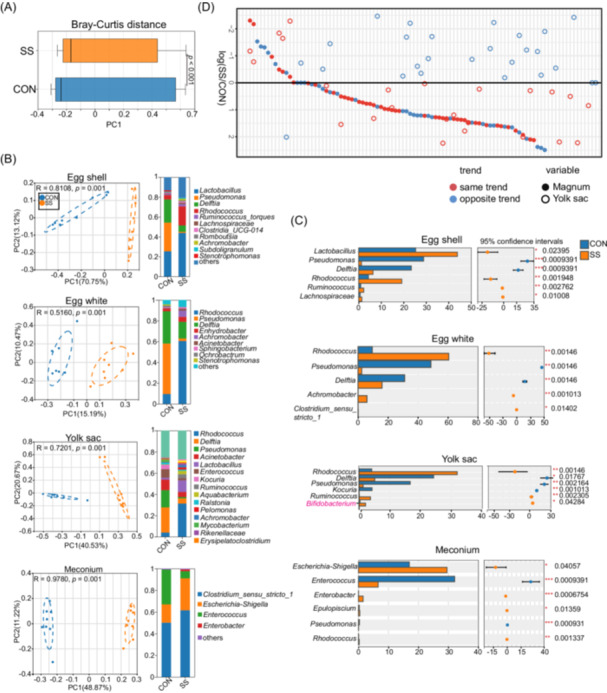
Offspring microbiota associated with maternal supplemented with soyasaponin (SS). (A) Bacterial community dissimilarities between offspring microbiota. Bray–Curtis distance was calculated for SS and CON samples independently. (B) PCoA of the unweighted UniFrac distances for egg shell, egg white, E19 yolk sac, and meconium microbiota, and relative abundance of different sites in genus level. (C) The most abundant genera with a significant difference between SS and CON groups. (D) Concordance of OTU variations between yolk sac and maternal magnum microbiota. The average relative abundance of the top 100 most abundant OTUs was compared between CON and SS groups. OTU, operational taxonomic unit; PCoA, principal coordinates analysis.

Investigating the influence of SS on chicken offspring, we analyzed Bray–Curtis distances in the microbiomes of egg white, yolk, and meconium samples, revealing SS significant impact on the vertical transmission of maternal microbiota (Figure 4A). PCoA analysis combined with genus‐level abundance comparison revealed distinct differences in microbial composition between the SS and CON groups across multiple embryonic compartments (Figure 4B). Dietary SS notably altered the shared bacterial genera between mothers and offspring and within different embryonic parts (Figure 4C). While embryonic stage microbial communities showed an influence on the intestinal microbiota structure at 7 days, Partial least squares discriminant analysis (PLS‐DA) analysis highlighted a distinct separation between the treatment groups (Figure S6). Particularly, the yolk sac and maternal magnum microbiota displayed significant concordance in microbial variation (Figure 4D).

### Dietary soyasaponin supplementation in maternal promotes chick development by reshaping microbiota

**FIGURE 5:**
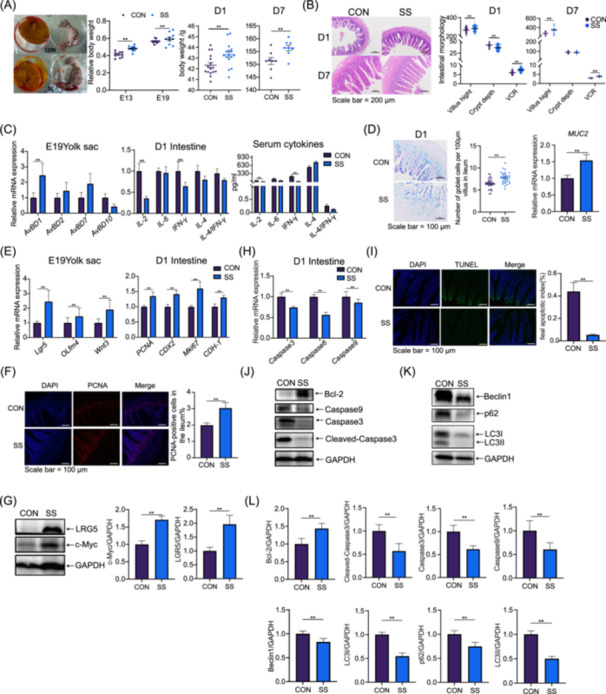
The embryonic stage is a key node for maternal nutritional intervention. (A) Relative embryo weight at E13, E19 (*n* = 10), average body weight of 1‐day‐old chicks (*n* = 16) after hatching, and 7‐day‐old chicks (*n* = 8). Data are shown as mean ± SEMs. (B) Morphological images of H&E‐stained ileum. Measurement of villus height (VH), crypt depth (CD), and villus to crypt ratio (VCR) at D1 and 7 (*n* = 64), bar = 200 μm. VH and CD measurements were obtained from 8 chicks in each group. Data are shown as mean ± SEMs. (C) Markers of embryonic immune development, including E19 yolk sac membrane Avian β‐Defensin (*AvBD)* mRNA expression (*n* = 8), serum cytokine levels (*n* = 8), and mRNA expression levels of cytokines in the ileum at 1 day (*n* = 8). Data are shown as mean ± SEMs. (D) Morphological images of AB‐PAS‐stained ileum. Count of goblet cells (*n* = 32) in the villus, bar = 100 μm. Data are shown as mean ± SEMs. Villus measurements were obtained from 8 chicks in each group. (E) E19 yolk sac membrane and 1‐day ileal mRNA expression levels related to proliferation and differentiation (*n* = 8). Data are shown as mean ± SEMs. (F) Representative immunofluorescence images of proliferating cell nuclear antigen (PCNA) in the ileum of 1‐day‐old chicken offspring. Ratio of PCNA‐positive cells in the ileum crypt and villus on day 1 (*n* = 8), bar = 100 μm. Data are shown as mean ± SEMs. (G) Western blot analysis of Leucine‐rich Repeat‐containing G‐protein Coupled Receptor 5 (LGR5), Cellular Myelocytomatosis Oncogene (c‐Myc) in the ileum on day 1 (*n* = 6). Data are shown as mean ± SEMs. (H–J) Representative images of the TUNEL assay in the ileum of 1‐day‐old chicken offspring. Statistical analysis of the apoptotic index in the ileum (*n* = 8), bar = 100 μm. Relative mRNA expression of caspase3, 6, and 9 in the 1‐day‐old chick's ileum. Western blot analysis of B‐cell lymphoma 2 (Bcl‐2), caspase 9, and caspase3 in the ileum on Day 1 (*n* = 6). Data are shown as mean ± SEMs. (K, L) Western blot analysis of Beclin‐1 Autophagy Related (Beclin1), Sequestosome 1 (p62), Microtubule‐associated Protein 1 A/1B‐light Chain 3 (LC3) in the ileum on day 1 (*n* = 6). Data are shown as mean ± SEMs. H&E, hematoxylin and eosin.

Dietary SS impact on maternal and offspring microbiomes across multiple body sites led to an investigation of its effects on chick development and maternal nutrient transfer. In broiler chickens, where the embryonic stage is crucial for later growth efficiency, SS notably increased the relative embryo weight at E13 and E19 stages, as well as the average body weights at 1 day and 7 days post‐hatch, compared to the control group (Figure 5A). Given the rapid intestinal development during early growth, the enhancement of VH and VCR, along with reduced CD in SS‐supplemented groups (Figure 5B), underscores the importance of early intestinal development. Specifically, Dietary SS supplementation significantly upregulated Avian beta‐defensin 1 (*AvBD1*) expression in the yolk sac membrane at E19 (Figure 5C), a key factor in early development and immune system maturation. In 1‐day‐old chicks, SS led to decreased serum IL‐2 levels and Interferon‐gamma (IFN‐γ), mirroring changes in mRNA expression levels in the ileum (Figure 5C). Additionally, SS enhanced intestinal immune function by significantly increasing the number of jejunal goblet cells and upregulating Mucin 2 (*MUC2*) mRNA expression levels (Figure 5D).

Dietary SS notably elevated the mRNA expression levels of key regulatory genes Leucine‐rich repeat‐containing G‐protein coupled receptor 5 (*Lgr5*), Olfactomedin 4 (*Olfm4*), and *Wnt3* (Figure 5E) involved in promoting cellular proliferation and differentiation of the yolk sac, thereby contributing to the expansion and functional specialization of this critical embryonic structure. Intestinal development is accompanied by proliferation, differentiation, apoptosis, and autophagy of bacteria. Therefore, marker proteins Microtubule‐associated protein 1 A/1B‐light chain 3 (LC3), B‐cell lymphoma 2 (Bcl2), and LGR5 were selected for immunofluorescence identification. The results showed that in the intestines of 1‐day‐old chicks, LC3‐positive cells significantly decreased in the SS group, while Bcl2‐positive and LGR5‐positive cells significantly increased in the SS group (Figure S7). Dietary SS promoted the expression of proliferation‐related genes including *PCNA*, Caudal‐type homeobox transcription factor 2 (*CDX2*), Marker of proliferation Ki‐67 (*Mki67*), and E‐cadherin (*CDH*‐1) (Figure 5E), alongside a significant increase in PCNA‐positive cells in the ileum (Figure 5F), indicating enhanced intestinal epithelial cell proliferation and differentiation. This was further supported by increased expression of marker proteins LGR5 and Cellular Myelocytomatosis Oncogene (c‐Myc) (Figure 5G). Dietary SS also played a role in regulating apoptosis and autophagy in intestinal development. It significantly suppressed mRNA levels of apoptosis‐related genes *caspase3*, *caspase6*, and *caspase9*, and enhanced Bcl‐2 protein expression, inhibiting caspase3 and Cleaved‐caspase3 (Figure 5H–J). Moreover, dietary SS reduced expression levels of key autophagy‐related proteins Beclin‐1 autophagy‐related (Beclin1), p62, LC3I, and LC3II (Figure 5K,L). These findings suggest that maternal SS supplementation promotes intestinal development in chicks by encouraging cell proliferation and differentiation while concurrently inhibiting apoptosis and autophagy pathways.

### Alterations in maternal microbial transmission via soyasaponin supplementation result in increased gamma‐aminobutyric acid in offspring intestinal microbiota

**FIGURE 6:**
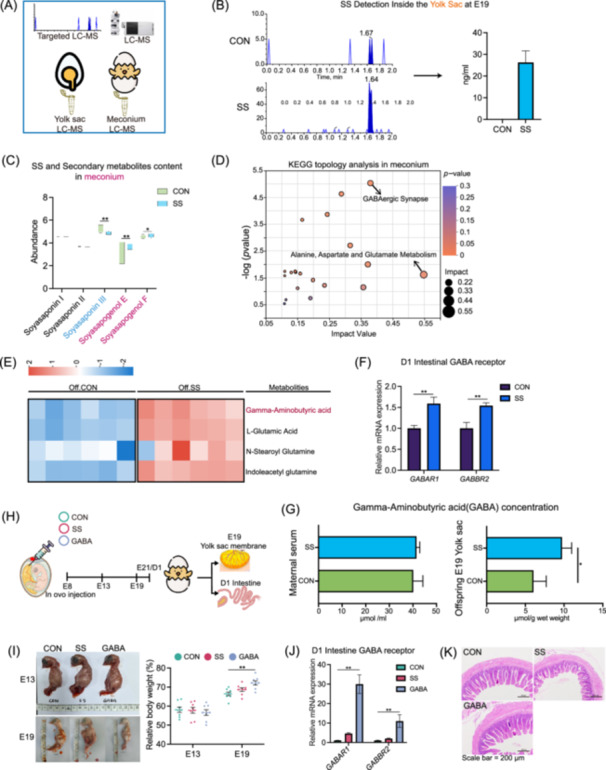
Dynamic changes in the content of SS in the yolk‐meconium axis and identification of characteristic metabolites in the meconium. (A) Flowchart of the identification process for the dynamic changes of soy saponins. (B) The concentration of SS deposited in the egg yolk (before incubation) following SS supplementation was determined using liquid chromatography‐mass spectrometry (LC‐MS). (C) Nontargeted LC‐MS detection of various SS configurations and their metabolite abundance in meconium (just after hatching). (D) Results of the KEGG topology analysis on differential metabolites in meconium. The size of the dots indicates the number of differential metabolites. (E) Screening of differential metabolites based on KEGG enrichment results combined with variable importance in projection (VIP) determination. (F) Relative mRNA expression of Gamma‐aminobutyric acid (GABA) receptors in the ileum at Day 1 (*n* = 8). Data are shown as mean ± SEMs. (G) Detection of GABA content in the serum of breeder chickens and the contents of the yolk sac at embryonic Day 19 (E19) (*n* = 8). Data are shown as mean ± SEMs. (H) Flowchart of the chicken embryo injection experiment. (I) Relative embryo weight at E13 and E19 (*n* = 8). Data are shown as mean ± SEMs. (J) Relative mRNA expression of genes related to GABA receptors in the D1 intestine (*n* = 8). Data are shown as mean ± SEMs. (K) Morphological images of H&E‐stained ileum, bar = 200 μm. H&E, hematoxylin and eosin.

Our research indicates that SS content in yolks of the SS‐treated group reached 26.25 ng/mL. Metabolomic analysis showed clear variations in soyasaponin metabolites in meconium between SS‐supplemented and control groups (Figure 6A,B). Specifically, the SS group exhibited lower Soyasaponin III and higher Soyasapogenol E levels, along with a rising trend of Soyasapogenol F (Figure 6C). These findings suggest the transmission of bacteria capable of metabolizing SS to the intestines of chicks. Differential metabolite enrichment analysis identified 655 unique metabolites, demonstrating clear separation in PLS‐DA groups (Figure S8A,B). Identified in the meconium, as annotated using the Human Metabolome Database (HMDB) database, were predominantly concentrated in the categories of lipids and lipid‐like molecules, as well as organic acids and derivatives (Figure S8C). Key findings include the enrichment of metabolites in the GABAergic Synapse and Alanine, Aspartate, and Glutamate metabolism pathway (Figure 6D), notably gamma‐aminobutyric acid (GABA), which is significantly increased in the SS group and crucial for fetal development (Figures 6E, S8D). To investigate whether the differential metabolite GABA exerts functional effects, we further measured the expression levels of Gamma‐aminobutyric acid type A receptor subunit alpha1 (*GABAR1*) and Gamma‐aminobutyric acid type B receptor subunit 2 (*GABBR2*) in the ileum of 1‐day‐old chick offspring (Figure 6F). To clarify the specific source of GABA, we measured the GABA content in the serum of breeder chickens and the contents of the yolk sac at E19. The results indicated that GABA showed no significant differences in the maternal group, but the yolk sac content was significantly increased in the SS group (Figure 6G).

### Soyasaponin efficacy dependent on vertically transmitted maternal microbial communities

To elucidate the maternal effects of SS, controlled injections of SS and GABA were administered to eggs in the control group. This approach aimed to ascertain the functionality of SS without the influence of SS‐modified microbial populations and to discern the end products responsible for its biological activity (Figure 6H). GABA injection significantly enhanced E19 embryos' relative body weight, though no notable impact was seen on 1‐day‐old chicks' average body weight (Figures 6I, S9A). The GABA treatment group showed activation of *GABRA1* and *GABBR2* expression, whereas the SS injection group did not (Figure 6J). GABA injection improved the intestinal VCR (Figures 6K, S9B). mRNA analysis in E19 yolk sac membranes showed GABA elevated expression of developmental markers like *Lgr5*, *Olfm4*, *Wnt3*, and various *AvBDs* (Figure S9C). GABA also upregulated mRNA of proliferative markers in 1‐day‐old chick intestines (Figure S9D). These findings suggest SS developmental benefits are mediated through metabolites formed after reshaping maternal microbiota. Further research is required to identify the specific bacteria involved in this interaction.

### Soyasaponin functions under the influence of vertically transmitted *Bifidobacterium adolescentis* in the maternal microbiota

**FIGURE 7:**
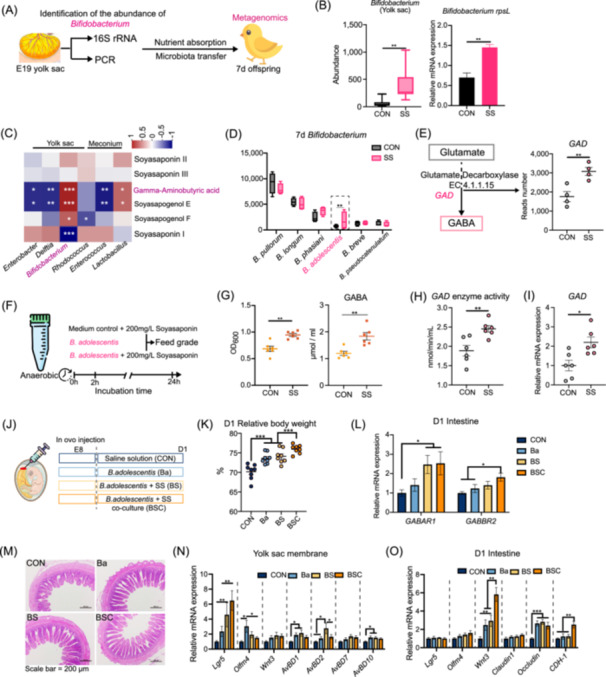
Increase of *Bifidobacterium* in offspring following maternal supplementation with SS, and the promotion of *Bifidobacterium adolescentis* proliferation by SS, resulting in the production of GABA. (A) Identification of *Bifidobacterium* in egg yolk contents and its transmission to the intestinal tract of 7 d chicks. (B) Identification of *Bifidobacterium* within the yolk sac. (C) Correlation analysis of differential bacteria in the yolk sac and meconium with secondary metabolites of SS and GABA in the meconium. (D) Identification of *Bifidobacterium adolescentis* (*B. adolescentis*) as a differential bacterium in the gut microbiome of 7‐day‐old chicks using metagenomics. (E) Glutamine metabolism generating GABA via Glutamate decarboxylase (GAD) enzyme based on 7‐day‐old intestinal microbiome metagenomic annotation (*n* = 4). Data are shown as mean ± SEMs. (F) Isolation of *B. adolescentis* and cocultivation with SS. (G) Coculture results of SS and GABA content (*n* = 6). Data are shown as mean ± SEMs. (H, I) Activity of Glutamate decarboxylase (GAD) enzyme and GAD mRNA expression levels in *B. adolescentis* after coculturing with SS for 24 h (*n* = 6). Data are shown as mean ± SEMs. (J) Injection of *B. adolescentis* (Ba), *B. adolescentis* + SS (BS), *B. adolescentis* + SS co‐culture (BSC) into chicken embryos. (K) Relative body weight on D1 (*n* = 8). Data are shown as mean ± SEMs. (L) D1 intestinal GABA receptor mRNA expression levels (*n* = 8). Data are shown as mean ± SEMs. (M) Intestinal villus morphology, bar = 200 μm. (N, O) Expression levels of mRNAs related to yolk membrane, intestinal proliferation, differentiation, and immune function (*n* = 8). Data are shown as mean ± SEMs.

Previous research has discovered that GABA production occurs during the embryonic stage. *Bifidobacterium*, identified as a key GABA‐producing bacterium through correlation analysis and literature review, showed consistent abundance in E19 yolk sac contents via PCR and gel electrophoresis, aligning with 16s rRNA results (Figures 7A–C, S10A). To ascertain the transmission and colonization of *Bifidobacterium*, we selected the intestinal microbiota of 7‐day‐old chick offspring for metagenomic analysis. At the species level, the SS group exhibited 1160 unique bacterial species, primarily associated with functions related to carbon metabolism, the AMPK signaling pathway, and the autophagy pathway was markedly downregulated in the SS group (Figure S10B–E). Additionally, the concentration of *Bifidobacterium adolescentis* (*B. adolescentis*) significantly increased in the SS group (Figure 7D). A trend towards increased GABAergic synapse pathways was also observed (Figure S10F). Further KEGG annotation revealed that the enzyme glutamate decarboxylase (GAD), which catalyzes the conversion of l‐glutamate to GABA, was significantly upregulated in the SS group (Figure 7E). To investigate whether this bacterium interacts with SS to metabolize and produce GABA, an in vitro coculture experiment with feed‐grade SS (primarily soyasaponin Ba) was performed (Figure 7F). After 24 h of coculture, enhanced growth of *B. adolescentis* and increased GABA content in the supernatant were observed in the presence of SS (Figure 7G). Both the enzyme activity and expression levels of GAD were significantly upregulated in the SS coculture group (Figure 7H,I). Further coculture experiments using pure Soyasapogenol E and F with *B. adolescentis* showed no promotion of bacterial growth. In contrast, Soyasaponin Ba demonstrated growth‐promoting effects similar to those observed with feed‐grade SS (Figure S11A).

To further validate the interaction between maternal vertical transmission of *B. adolescentis* and the deposited SS in the yolk sac, we performed an injection experiment in chicken embryos (Figure 7J). The gel electrophoresis of *Bifidobacterium*‐specific primers was first performed by extracting fetal fecal DNA, which showed more distinct bands in the Ba group compared to the BS group (Figure S11B). Both Ba and BS groups significantly increased the relative average body weight of D1 chicks (Figure 7K). Among them, mRNA expression of *GABAR1* in the intestine was significantly upregulated in the BS and BSC groups, while *GABBR2* was significantly upregulated in the BSC group (Figure 7L), indicating activation of GABA receptors. The VH of the Ba group, BS group, and BSC group were all significantly elevated, with the BS group outperforming the Ba group (Figures 7M, S11C). In the yolk membrane, the mRNA expression of *Lgr5*, *olfm4*, *AvBD1*, *AvBD2*, and *AvBD10* was significantly upregulated in the Ba, BS, and BSC groups, with the BS and BSC groups showing more pronounced effects (Figure 7N). In the intestines of D1 chicks, *Wnt3*, *Occludin*, and *CDH‐1* were significantly upregulated in the BSC group (Figure 7O).

## DISCUSSION

The critical role of effective vertical transmission of maternal microbiota in fetal development is underscored in our study. While the transfer of microbiota from the chicken reproductive tract to offspring via egg white is acknowledged, the dynamics of intestinal transmission and the duration of its influence remained less explicit. We conducted a spatiotemporal analysis of avian multi‐site flora and intervened with soybean saponins to comprehend how maternal flora transfer affects offspring development.

The cloaca, a unique anatomical junction of intestinal and reproductive tracts in birds, serves as a central hub for microbial transmission [18]. Previous research suggests limited microbial community transmission in birds, primarily influenced by the nest and maternal feather microbiota [19]. The eggshell most closely reflects the maternal intestinal microbiota, highlighting the gut microbiota as a primary target of dietary interventions. However, only a minor portion of eggshell flora transitions into the meconium, aligning with avian research findings [20]. Contrary to previous reports, a greater similarity was noted between the egg white and eggshell microbiota compared to the meconium composition [21], potentially due to the resemblance of reproductive tract microbiota and the core fungal genera in meconium. Dominant taxa such as *Enterobacteriaceae* and *Escherichia* in meconium samples align with mammalian findings, supporting vertical transmission from the reproductive tract [22]. Throughout the embryonic stage up to 7 days post‐hatch, the chick's primary nutritional source is the yolk sac [23]. Previous studies have demonstrated a progressive increase in microbial diversity within the yolk sac from embryonic Day 7 to Day 15, with relative abundance exceeding that of egg whites [24]. Studies have shown that injecting probiotics into the yolk sac is an effective method for regulating chicken embryo development. This suggests that the yolk sac has a stable microbial community structure and a certain level of adaptability to exogenous bacteria [25]. However, whether the rapid surge in gut microbial abundance observed in chicks during the first 1 to 7 days post‐hatching is associated with the yolk sac microbiota remains unclear [26]. The yolk sac is identified as a pivotal contributor to early microbiota development in chicks, evidenced by the marked similarity between the yolk sac and the gut microbiome of 7‐day‐old chicks. Key transmitted genera, including *Lactobacillus*, *Bifidobacterium*, and *Escherichia‐Shigella*, underscore the yolk sac's role in shaping the early gut microbiome [27]. The initial dominance of facultative anaerobes in the chick intestine post‐hatching aligns with the presence of these core genera, further affirming the yolk sac's significance in early microbial colonization.

Bacteria regulated by the host genotype are considered to have significant heritability [17]. This study, for the first time, performed a GWAS‐based association of transmitted bacteria in both maternal and offspring chickens, confirming that the core transmitted bacterium *Bifidobacterium* is regulated by colocalizing genetic variants in both the maternal and offspring. The *IMP3* gene, which regulates the signaling of growth factor *IGF2*, directly controls embryonic development [28]. Although many known transmitted bacteria, such as *Lactobacillus*, did not show significant colocalizing variants, near‐significant results were observed, which may be attributed to the relatively small sample size [29]. The significant variants colocalized with *Bifidobacterium* and *Lactobacillus* in the parents are primarily involved in regulating organismal development and immune function, which aligns with the known functions of these bacteria [30]. In conclusion, this study further demonstrates the transmissibility of microbiota through colocalizing variants in maternal and paternal genomes. However, the impact of nutritional interventions on the transmission of maternal microbiota remains largely unknown.

The known soybean bioactive factors presently exert maternal effects in breeder hens primarily through epigenetic mechanisms, reshaping of gut microbiota, antibody transmission, and nutrient deposition in the offspring [31]. SS assumes a prebiotic role when incorporated into low‐soybean meal diets and when in synergy with the gut microbiota [32]. Our results suggest that SS supplementation in chicken increased the relative abundance of both maternal gut microbiota and reproductive tract microbiota, increasing the number of genera shared by different sites. The specific physiology of the cloaca connects the intestinal tract to the reproductive tract, and bacteria can be transferred in this pattern, which is one of the most important reasons affecting the reproductive performance of laying hens. Notably, two distinctive shared genera, *Rhodococcus* and *Pseudomonas*, consistently display changes in relative abundance across various sites. *Pseudomonas*, a potential pathogen, can exert an impact on the health of the intestinal and reproductive tracts [33]. It is indicated that SS, under the influence of the gut microbiota, enhances laying performance and immune function in laying hens [14]. This activation promotes B cell maturation and differentiation, ultimately improving immune function in broilers [34]. This horizontal mode of transmission of SS‐promoting bacteria in the intestinal‐reproductive tract improves immune function in breeder hens. The regulatory mechanisms involving in the vertical transmission for offspring growth and development need to be further investigated.

Our study demonstrates the significant impact of SS on the vertical transmission of maternal microbiota, highlighting a convergence in microbial community structures across various body sites in SS‐supplemented breeder hens and their offspring. Notably, genera such as *Rhodococcus*, *Delftia*, *Clostridium*, and *Ruminococcus*, known for fostering early intestinal microbial maturation [35], showed substantial increases in relative abundance. The hatching period is crucial for establishing host‐microbe interactions and is essential for balanced immune development [23]. Unlike direct antibody transfer, the maternal microbiome indirectly nurtures fetal immune development. Maternal SS supplementation significantly amplifies the mRNA expression of *AvBD1* in the yolk sac membrane at the E19 embryo stage, facilitating the maturation of intestinal goblet cells and promoting intestinal development. Due to the oviparous nature of poultry, maternal antibodies become fixed upon transmission to the eggs [36]. Therefore, *AvBD* secreted by the yolk sac membrane serves as the primary antimicrobial substances during the early development of chicken embryos [37], and their strength reflects the early embryonic immune development level. Previous studies have shown that maternal IgG antibodies, bound to microbial molecules and transmitted to the offspring, play a crucial role in postnatal innate immune development [38].

Delayed intestinal development in chicks is caused by *in ovo* nutritional deficiencies during embryonic development [39]. A previous study found that inactivation of mTOR and activation of the autophagy protein LC3 by undernutrition of the intestinal epithelium during the late stages of embryonic development is the main mechanism [40]. We discovered that maternal SS supplementation significantly boosts the mTOR precursor arginine synthesis metabolic signaling pathway. This process is marked by elevated expression of Bcl‐2, inhibiting the activation of p62 with LC3, and suggesting the potential role of SS in regulating autophagy within intestinal epithelial cells. Overall, our results show that maternal SS supplementation affects the vertical transmission of bacteria and metabolic processes, as well as interactions with the development of the offspring's intestinal tract.

Another discovery in this study is that the yolk sac can also serve as a carrier for the transmission of SS and bacterial metabolites. In the meconium, we observed a significant decrease in the amount of soy saponin III, which is a major component of SS. In contrast, its metabolites, Soyasapogenol E and F, displayed a significant increase or a rising trend. These findings align with earlier investigations, indicating that SS requires the presence of specific bacterial genera in the gut for metabolism into simpler substances, thereby enabling its functionality [32]. Given that bacterial metabolites can also be transferred to meconium, we identified a marked elevation in the GABA content, a vital substance for early fetal neurodevelopment and intestinal maturation [41]. Research suggests that GABA primarily originates from the metabolic activities of the gut microbiota [42]. During the fetal stage, the primary bacteria responsible for GABA production are *Escherichia coli*, *Bacteroides*, and *Bifidobacterium* [42]. GABA plays a pivotal role in promoting intestinal neurodevelopment in the fetal gut by activating GABA receptors, *GABRA1* and *GABBR2*, which in turn stimulate potassium‐calcium ion channels [43]. We next sought to determine the key role of GABA receptor activation. This examination uncovered a notable expression of GABA receptor genes in the ileum of 1‐day‐old chicks. To ascertain the specific substance influencing early embryonic development, an *in ovo* injection experiment was conducted, utilizing SS and GABA on fertilized eggs.

It was found that GABA significantly increased the average weight of E19 embryos and 1‐day‐old chicks. In addition, the injection of GABA into the embryos promoted the proliferation and differentiation of the yolk sac and intestine and enhanced immune function. In contrast, SS injections into chicken embryos had an inhibitory effect on intestinal villi development, which contradicts prior research findings and reports. SS may need to work in conjunction with intestinal microbiota to function [32]. The results also suggest that SS alone cannot exert its function and may require the involvement of specific bacterial genera. Subsequent observations revealed that SS embryo injections did not activate GABA receptors in the chick's intestines. These findings indicate that without the specific transmission of maternal microbial genera, SS cannot exert its prebiotic effect. Therefore, identifying bacteria that can interact with SS is essential for understanding how SS exerts maternal effects.

Previous research has indicated that *Bifidobacterium* influences development and immune function in the early stages of infant life through its metabolic products [44]. *Bifidobacterium* abundance significantly increased in breeders' magnum and E19 yolk sac contents. To further investigate whether *B. adolescentis* interacts with SS during early embryonic development, we analyzed *B. adolescentis* in association with GABA and SS secondary metabolites. It was found that *Bifidobacterium* is involved in the metabolism of these substances. To further verify that the yolk sac microbiota is a critical factor in the post‐hatch chick gut microbiota development, a metagenomic analysis of the gut microbiota in 7‐day‐old chicks was conducted. This analysis revealed a significant increase in the quantity of *B. adolescentis*. A recent study indicated that *B. adolescentis* is a key member of the human gut microbiota responsible for GABA production, and it can be shared between mothers and infants [45]. Next, we conducted an in vitro coculture study with *B. adolescentis* and SS, suggesting that SS increased the abundance of *B. adolescentis* at the end of the culture period. Additionally, a significant increase in the concentration of GABA in the culture supernatant was noted. This suggests that the maternal effect in poultry might involve interactions between nutrients and vertically transmitted microbial communities during the chicken embryo development window, which is pivotal for promoting intestinal development. Consequently, this indicates that the optimal timing for maternal microbial transmission to exert its effect is likely during this developmental window.

## CONCLUSION

In summary, our research elucidates that in birds, gut microbiota, in addition to reproductive tract microbiota, can be transferred to offspring chicks via the unique physiological structure of the cloaca. We have pinpointed the yolk sac's pivotal role in the vertical transmission of maternal microbiota and highlighted that these microbes are regulated by heritable host genotypes. This study utilized soyasaponin supplementation to probe the precise mechanisms of gut microbiota interactions with nutrients, affecting host gut maturation. Specifically, soyasaponin requires synergy with vertically transmitted microbiota to metabolize GABA, thereby inhibiting autophagy and apoptosis in intestinal epithelial cells and fostering cellular proliferation and differentiation, ultimately enhancing offspring chick development. Nonetheless, the intricate interplay between maternal nutritional deposition and vertical microbial transmission remains a complex field, with many aspects yet to be fully comprehended. Future research should aim to unravel the nuances of vertical transmission and its profound implications for vertebrate biology.

## METHODS

Broiler breeder hen feeding experiment and sampling A flow diagram of the experimental design is shown in Figure S12.

### Animals and experiment design

A total of 480 broiler breeders were randomly divided into two groups. A control group and a soya saponin (SS) supplementation group, each comprising eight replicates with 30 hens. The hens were housed in dual‐nippled drinker and feeder‐equipped cages, two per cage. Post 1‐week acclimatization, CON received a basal diet, while SS was administered the same diet augmented with 200 mg/kg soya saponin (45% purity, Xi'An TongZe Biotech Co., Ltd.) over 7 weeks. The diet adhered to AA Parent Substitute Breeder Chicken Nutrition standards (Table S6), with a daily feed restriction of 160 g.

In the final week, 240 eggs per group were incubated in 8 replicates of 30, in a single incubator without disinfection, to study microbial transmission. Eggs were distributed across 8 trays, with each tray holding one replicate per treatment. The incubator was pre‐disinfected and filled with sterile water, but eggs were intentionally not disinfected to focus on microbial transmission effects. Post‐hatch, one technician performed sex identification, and 160 1‐day‐old males (80 per group) were placed in controlled brood cages, reflecting maternal diets, in eight replicates of 10. Temperatures were maintained at 33–35°C with free access to a basal diet, adhering to AA Commercial Broiler Nutrition standards (Table S6).

An in *ovo* injection experiment was conducted at the Meat Chicken Science and Technology Backyard Program, Zhuozhou, Hebei. Ninety white‐feathered eggs from Huadu Yukou Breeding Farm (47 weeks old) were incubated and split into three treatment groups: 1% tween 80 (sterile), 1 µg soyasaponin per egg (SS), and 10 µg γ‐Aminobutyric Acid per egg (GABA), with six replicates of five eggs each. On Day 10 of incubation, a small hole was drilled at the blunt end of each egg, and solutions of SS (40% purity, Xi'An TongZe Biotech) and GABA (98% purity, Shanghai Yuanye Bio‐Technology) in 1% tween80 were injected into the yolk (100 μL per egg). In a separate experiment, four treatment groups were established. The control group was injected with physiological saline (CON). One group received 1 × 10^2^ *B. adolescentis* (Ba). Another group was administered a mixture of 1 × 10^2^ *B. adolescentis* and 1 µg soyasaponin. The final group was injected with a diluted product (BSC) obtained by coculturing *B. adolescentis* and soyasaponin for 24 h. The entire *in ovo* injection procedure was conducted under a laminar flow hood to minimize interference from environmental microorganisms. The eggs were then sealed with tape and returned to the incubator. Fertilization rates were assessed by candling on Day 16, and the number of dead embryos was recorded on the hatching day.

### Sample collection

At the conclusion of the hen feeding trial, eight hens from each group were randomly selected and euthanized for sampling. Serum was collected by centrifuging blood from the wing vein at 3000 rpm for 15 min, then stored at −80°C for immune factor analysis. The central ileum section was preserved in 4% paraformaldehyde, while the remaining ileum was stored at −80°C for future investigations. Cecal digesta, magnum, and cloaca swabs were collected in sterile tubes and frozen in liquid nitrogen for bacterial 16S rRNA gene sequencing. Magnum tissue was collected in RNase‐free tubes and stored at −80°C for subsequent qPCR assays.

To minimize contamination from feathers, cage conditions, and eggshell‐associated microorganisms, microbial samples were collected immediately after egg laying. Eggshells were swabbed upon collection, and eight eggs from each group were selected for 16S rRNA gene sequencing to assess microbial communities. Before incubation, egg whites and yolks from these eggs were separated in a vertical flow clean bench and stored at −80°C, intended for analyzing SS deposition in yolks and sequencing the egg whites. On embryonic Days 13, 19, and hatching day, one normal embryo per replicate (8 per group) was chosen, with all sampling performed under sterile conditions. Embryonic development was assessed by weighing the embryos. Samples from the yolk sac membrane and yolk sac were collected aseptically and stored at −80°C for qPCR and sequencing. At hatching and at 7 days old, 8 chicks per group were randomly selected and euthanized after weighing. Blood was drawn, with serum stored at −80°C. Ileum, cecum, and yolk sac membrane samples were collected; a 1 cm section of the middle ileum was fixed in 4% paraformaldehyde, with the rest stored at −80°C. Meconium from newborns and cecal digesta from 7‐day‐old chicks were preserved in liquid nitrogen for sequencing, liquid chromatography‐mass spectrometry (LC‐MS), and metagenomic analysis.

The sample collection of the *inovo* injection experiment was performed on the hatching day. We randomly selected 8 chickens from the CON, SS, and GABA groups for the sample collection. The hatching weight was measured. After euthanasia, the yolk sac and ileum were carefully collected and stored at −80°C. The ileum was fixed in 4% paraformaldehyde.

### Determination of serum hormone levels, immune indexes

The concentrations of Follicle Stimulating Hormone (FSH), Luteinizing Hormone (LH), and Estradiol (E2) in serum were measured using a radioimmunoassay kit from Beijing Northern Biotechnology Institute, China. Additionally, interleukin‐2 (IL‐2), IL‐6, IL‐4, and interferon‐γ (IFN‐γ) levels were quantified in the serum of both breeders and newly hatched chicks using a protocol from Beijing Solarbio Science & Technology Co., Ltd., China, adhering to the manufacturer's instructions.

### Morphology analysis

Adhering to the methodology delineated by Gao [13], the ileal sections from breeders, 1‐day‐old, and 7‐day‐old chicks were fixed in 4% paraformaldehyde for a duration of 24 h. Following fixation, the sections were treated sequentially with a graded series of ethanol and xylene, culminating in paraffin embedding. The sections were then deparaffinized in xylene and rehydrated through graded ethanol dilutions. Hematoxylin and Eosin and Alcian Blue‐periodic acid Schiff staining were subsequently performed. Goblet cells, intestinal villi, and crypts in the intestinal sections were quantified using the method described by Jiang [46].

### Immunohistochemistry

Immunohistochemistry on ileum sections included deparaffinization, hydration, heat‐induced antigen retrieval, and blocking of endogenous peroxidase activity, following the method detailed by Jiang [47]. The PCNA antibody (Abcam) and LC3 (Abclone; A19700) were used for the immunofluorescence assay. The TUNEL assay, using a TUNEL BrightGreen apoptosis detection kit (Vazyme Biotech), assessed ileal apoptosis. Quantitative analysis was conducted using Image‐J software. The ratio of proliferating cells (red) to total cells (blue) indicated positive cells, and the ratio of apoptotic cells (green) to total cells (blue) quantified the apoptotic index.

### Gene expression measurement and analysis

A 100 mg tissue sample was placed into 1 mL of Trizol (Vazyme Biotech) for total RNA isolation, quantification, cDNA synthesis, and real‐time PCR, following the protocol described by Lv [48]. Gene expression analysis was performed via RT‐PCR using primers listed in Appendix Table S7 and ChamQ SYBR qPCR Master Mix (Vazyme Biotech) on an Applied Biosystems 7500 Fast Real‐Time PCR System. The reaction volume was 20 μL. Relative gene expression levels were determined using the 2^−ΔΔCt^ method, with normalization against β‐actin.

### Western blot analysis

Western blot analysis was used to detect PCNA (Abcam; ab29), c‐Myc (Jingjie; PTM‐5143), LGR5, caspase3, 9, Bcl‐2, p62, LC3, and Beclin1 (Abclone; A12327, A25309, A11451, A18415, A19700, and A12319) levels in the newly hatched chick's ileum. Total protein was extracted from each sample using RIPA buffer(high) (Solarbio Science; R0010) supplemented with PSMF (Solarbio Science; P0100). According to the manufacturer's protocol, the total protein content was determined using the BCA Protein Quantification Kit (Vazyme Biotech). Equal amounts of protein (10 mg) were subjected to electrophoresis and then transferred onto a polyvinylidene difluoride membrane (Millipore). Finally, the blot was washed, and protein bands were detected using the ECL Western blot analysis substrate (Solarbio Science; PE0010). Signal visualization was performed using the ChemiDoc Touch Imaging System (Bio‐Rad Laboratories).

### 16s rRNA gene sequencing and analysis

Sequencing and analysis according to the method described by Gao [49]. Microbial genomic DNA was isolated utilizing the DNA extraction kit (QIAamp Fast DNA Stool Mini Kit, Qiagen Company), following the guidelines provided by the manufacturer. Universal primers 338F (5′‐ACTCCTACGGGAGGCAGCA‐3′) and 806R (5′‐ GGACTACHVGGGTWTCTAAT‐3′) targeting the V3‐V4 region of the 16SrDNA gene were employed for the amplification of bacterial DNA. All samples were processed in the same batch, including negative controls (sterile water) to detect potential contamination from reagents and the reaction system. The cleanliness of reagents and the environment was assessed by monitoring whether amplification products were present in the negative controls. High‐purity reagents free of DNase, such as TransStart Fastpfu DNA Polymerase (TransGen AP221‐02), were used. All reagents were newly opened to avoid contamination due to prolonged usage. On‐machine sequencing was performed using HiSeq. 2500 PE250. Sequencing analysis was carried out by Shanghai Major Bio‐Information Technology Co., Ltd. The generation of species abundance tables at various taxonomic levels was accomplished using Qiime software (Qiime2–2019.7; Nature Biotechnology). Additionally, LEfSe analysis was performed to identify biomarkers with statistically significant differences between groups based on the LDA value [50]. Venn diagrams and PCoA plots were performed using the online platform of Majorbio Cloud Platform. These networks were meticulously constructed utilizing the Fruchterman‐Reingold layout algorithm, incorporating 104 permutations, as facilitated by the igraph package in R (version 3.6.3).

We explored the early acquisition and development of microbiomes in the offspring's egg shell, egg white, yolk sac, meconium, and 7‐day‐old intestinal sites, focusing on the impact of different maternal sources using a longitudinal, multi‐site 16S rRNA approach. Samples from most eggshells and neonates were collected immediately after laying to prevent in vitro microbial exposure and colonization, aiding in the establishment of the meconium microbiota. 16S rRNA gene sequencing detected bacterial presence across all embryonic body sites, with areas like the eggshell and yolk sac contents exhibiting high species richness. Analyses such as PCoA and Bray–Curtis dissimilarity demonstrated clear clustering by sample type, indicating microbial origins likely independent of maternal or reagent contamination.

### The association between maternal vertical transmission of microbiota and host genotype

To clarify the association between host genetic kinship and microbial composition similarity based on pairs of individuals, we first calculated the genetic relationship matrix based on all SNP genotypes using GCTA (v.1.94.1). DNA was extracted from 88 blood samples using the CTAB method. Sequencing libraries were constructed using the Truseq Nano DNA HT Sample Prep Kit (Illumina) according to the manufacturer's instructions, with index codes assigned to each sample. Trimmed reads were aligned to the chicken reference genome (bGalGal1.pat.whiteleghornlayer.GRCg7w) using the BWA‐MEME software under default mapping parameters. Before further analysis, several filtering steps were performed to minimize false positives in SNP/InDel detection and genotype calling. First, the degree of relatedness (kinship) among all individuals was estimated using the Plink software [51], based on pairwise comparisons of SNP data [52]. Genome‐wide association studies (GWAS) were conducted for the target traits using the “rMVP” R software package with the FarmCPU model [53]. The first principal components (PCs) and the kinship matrix were calculated and incorporated as covariates in the GWAS model to account for population structure and relatedness.

### Metagenomic sequencing of cecal microbiota

Metagenomic sequencing of 7 d chicken's cecal microbiota DNA involved the extraction of DNA from cecal content using the QIAamp Fast DNA Stool Mini Kit (51604; Qiagen). The library underwent quality inspection, and those passing the inspection were sequenced on an Illumina HiSeq platform at the Shanghai Major Bio‐Information Technology Co., Ltd. The library was constructed using the NEXTFLEX Rapid DNA‐Seq kit (Bioo Scientific) through adapter ligation, magnetic bead selection to remove self‐ligated fragments, PCR amplification, and magnetic bead recovery of the final library. Sequencing was performed on the Illumina NovaSeq platform (Illumina) using bridge PCR. Raw reads were filtered to obtain high‐quality clean reads, ensuring the accuracy of subsequent analyses. SOAPdenovo2.53 was employed for assembly to generate scaftigs. MetaGeneMark [54] was used for gene prediction based on effective scaftigs, constructing a gene catalog. Subsequent analyses involved studying the composition or differences in species between samples based on clean reads using MetaPhlAn2 (v. 2.6.0) software [55].

### LC‐MS detection of the content of soybean saponins in egg yolks

The chromatography and mass spectrometry analyses were performed using UPLC (SHIMADZU, Nexera, X2) and MS/MS (Applied Biosystems, 4500, QTRAP), respectively. Substance identification relied on secondary mass spectrometry data from the MetWare Database (MWDB) for qualitative analysis. The triple quadrupole mass spectrometer's multiple reaction monitoring (MRM) mode was used for quantification. Analyst 1.6.3 software processed the mass spectrometry data, generated MRM metabolite detection chromatograms, and analyzed the total ion chromatograms to identify the SS product.

### Metabolic profiling of the newly hatched chick's meconium

Due to the elevated lipid content found in the yolk sac, we extended our investigation to perform a nontargeted metabolomic analysis on meconium for the identification of distinct bacterial metabolites. Meconium metabolites were subjected to untargeted metabolomics analysis using an Agilent 1200 series high‐performance liquid chromatography system (Agilent Technologies). The metabolites of serum at different time points were assessed using OPLS‐DA using the Majorbio Cloud platform (https://cloud.majorbio.com). (v1.24.0). The VIP was calculated, reflecting the loading weight and the response variability explained by this component. KEGG Pathway analysis of metabolites in each cluster was performed using the Majorbio Cloud platform.

### Assessment the impact of soya saponin on the growth of *B. adolescentis*

*Bifidobacterium adolescentis* was introduced into Reinforced Clostridium Medium, supplemented with 200 mg/L of soyasaponin (as determined in previous concentration gradient experiments), as well as 80 mg/L of soyasapogenol E, soyasapogenol F, or soyasaponin Ba (98% purity, Shanghai Yuanye Bio‐Technology) and incubated anaerobically for 24 h. Samples were obtained at the 24‐h mark, and the optical density at 600 nm was assessed using a microplate reader (BioTek Epoch2). Following centrifugation at 2000 rpm for 8 min, the resultant supernatant was utilized for GABA content analysis using the γ‐Aminobutyric Acid (GABA) Colorimetric Assay Kit E‐BC‐K852‐M (Elabscience Biotechnology Co., Ltd.), and GAD (EC 4.1.1.15) assay kit (JL‐T0864; Jonlnbio).

### Statistical analysis

Statistical analysis was conducted using SPSS 24.0. Unpaired two‐tailed Student's *t*‐test was employed to assess significant differences between the two groups. For datasets encompassing more than two groups, a one‐way analysis of variance was performed, followed by Duncan's multiple comparisons test. The presentation of data is in the form of means ± SEM. Significance levels were denoted as ** for *p* < 0.05, and *** for *p* < 0.01, indicating statistical significance.

## AUTHOR CONTRIBUTIONS

**Mingkun Gao**: Software; data curation; investigation; validation; formal analysis; writing—original draft; methodology; visualization. **Shu Chen**: Data curation; investigation. **Hao Fan**: Methodology. **Peng Li**: Methodology; investigation. **Aiqiao Liu**: Funding acquisition; methodology; resources. **Dongli Li**: Funding acquisition; methodology; resources. **Xiaomin Li**: Investigation; methodology. **Yongfei Hu**: Writing—review and editing; Conceptualization; methodology; supervision. **Guofeng Han**: Resources. **Yuming Guo**: Conceptualization; supervision; writing—review and editing; funding acquisition; project administration. **Zengpeng Lv**: Conceptualization; funding acquisition; writing—review and editing; project administration; supervision.

## CONFLICT OF INTEREST STATEMENT

The authors declare no conflicts of interest.

## ETHICS STATEMENT

The experimental protocols involving animals were authorized by the Animal Care and Use Committee of China Agricultural University (Approval No. AW01703202‐1‐6).

## Supporting information


**Figure S1.** Maternal microbes transfer to the eggshell and yolk sac.
**Figure S2.** The shared bacterial genera between maternal and offspring, as well as among different embryonic compartments.
**Figure S3.** The heritability and significant variants of the phenotype in maternal and offspring.
**Figure S4.** Differential bacteria in breeder chicken's intestine, magnum, and cloaca.
**Figure S5.** Impact of dietary soyasaponin supplementation on the health of the intestinal and reproductive tracts in broiler breeders.
**Figure S6.** Intestinal microbiota at 7 days in broiler chicken offspring.
**Figure S7.** D1 offspring intestinal immune fluorescence results.
**Figure S8.** Metabolomic structure of meconium.
**Figure S9.** In‐ovo injection results.
**Figure S10.** *Bifidobacterium* levels from embryonic stage to 7 days post‐hatch.
**Figure S11.** In‐ovo injection and co‐culture results.
**Figure S12.** Flow diagram.

**Table S1.** The number of SNP sites associated with shared bacteria between maternal and offspring.
**Table S2.** Identification of co‐localized variations in *Bifidobacterium* transmitted from maternal to offspring on chr3.
**Table S3.** Identification of co‐localized variations in *Bifidobacterium* transmitted from maternal to offspring on chr11.
**Table S4.** KEGG enrichment of genetic variants associated with *Lactobacillus*.
**Table S5.** KEGG enrichment of genetic variants associated with *Bifidobacteria*.
**Table S6.** Table composition and nutrient levels of basal diet (air‐dry basis).
**Table S7.** Sequences of the oligonucleotide primers used for quantitative real‐time.

## Data Availability

The data that support the findings of this study are openly available in NCBI at https://www.ncbi.nlm.nih.gov/, reference number PRJNA1077308. The 16S rRNA gene sequencing and Metagenomics sequencing data generated and analyzed during the current study are available in the NCBI primary data archive with accession number (PRJNA1077308, https://www.ncbi.nlm.nih.gov/bioproject/?term=PRJNA1077308). The metabolomics data reported in this paper have been deposited in the OMIX, China National Center for Bioinformation/Beijing Institute of Genomics, Chinese Academy of Sciences (OMIX009784, https://ngdc.cncb.ac.cn/omix/release/OMIX009784). The data and scripts for analysis and visualization are saved in GitHub https://github.com/erzhangao/mkiMeta. Supplementary materials (figures, tables, graphical abstract, slides, videos, Chinese translated version, and update materials) may be found in the online DOI or iMeta Science http://www.imeta.science/.
