# Supplementary material for: Soyasaponin and vertical microbial transmission: Maternal effect on the intestinal development and health of early chicks
Source: Imeta. 2025 May 20;4(4):e70044. doi: 10.1002/imt2.70044 (PMC12371269; doi:10.1002/imt2.70044)
Supplement: Supplementary file 1 — Figure S1. Maternal microbes transfer to the eggshell and yolk sac. Figure S2. The shared bacterial genera between maternal and offspring, as well as among different embryonic compartments. Figure S3. The heritability and significant variants of the phenotype in maternal and offspring. Figure S4. Differential bacteria in breeder chicken's intestine, magnum, and cloaca. Figure S5. Impact of dietary soyasaponin supplementation on the health of the intestinal and reproductive tracts in broiler breeders. Figure S6. Intestinal microbiota at 7 days in broiler chicken offspring. Figure S7. D1 offspring intestinal immune fluorescence results. Figure S8. Metabolomic structure of meconium. Figure S9. In‐ovo injection results. Figure S10. Bifidobacterium levels from embryonic stage to 7 days post‐hatch. Figure S11. In‐ovo injection and co‐culture results. Figure S12. Flow diagram. [file IMT2-4-e70044-s001.docx]

**Supporting information to**

**Soyasaponin and vertical microbial transmission: Maternal effect on the intestinal development and health of early chicks**

**Running title:** Soyasaponin alters chick microbiota transfer

Mingkun Gao^1^, Shu Chen^1^, Hao Fan^4^, Peng Li^1^, Aiqiao Liu^3^, Dongli Li^3^, Xiaomin Li^3^, Yongfei Hu^1^, Guofeng Han^5^, Yuming Guo^1^*, Zengpeng Lv^1,2^*

^1^State Key Laboratory of Animal Nutrition and Feeding, College of Animal Science and Technology, China Agricultural University, Beijing 100193, China

^2^China Agricultural University-Sichuan Advanced Agricultural & Industrial Institute, Chengdu 611430, China

^3^Beijing Huadu Yukou Poultry Industry Co., Ltd., Beijing 101206, China

^4^Department of Medicine, The University of Chicago, Chicago 60637, USA

^5^Institute of Agricultural Facilities and Equipment, Jiangsu Academy of Agricultural Sciences，Nanjing 210014, China

^*^Correspondence: lvzengpeng310@cau.edu.cn (Zengpeng Lv), guoyum@cau.edu.cn (Yuming Guo)


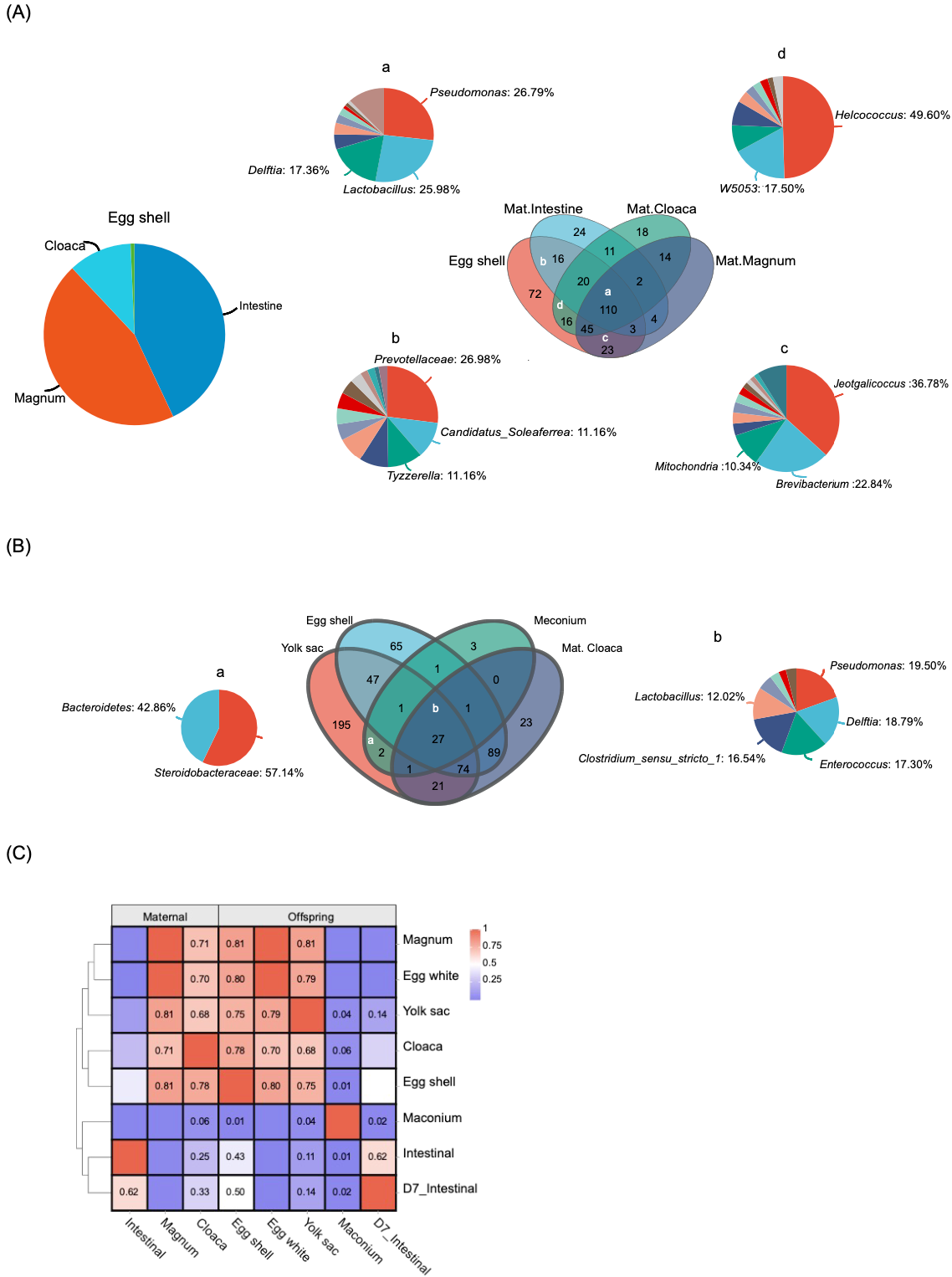

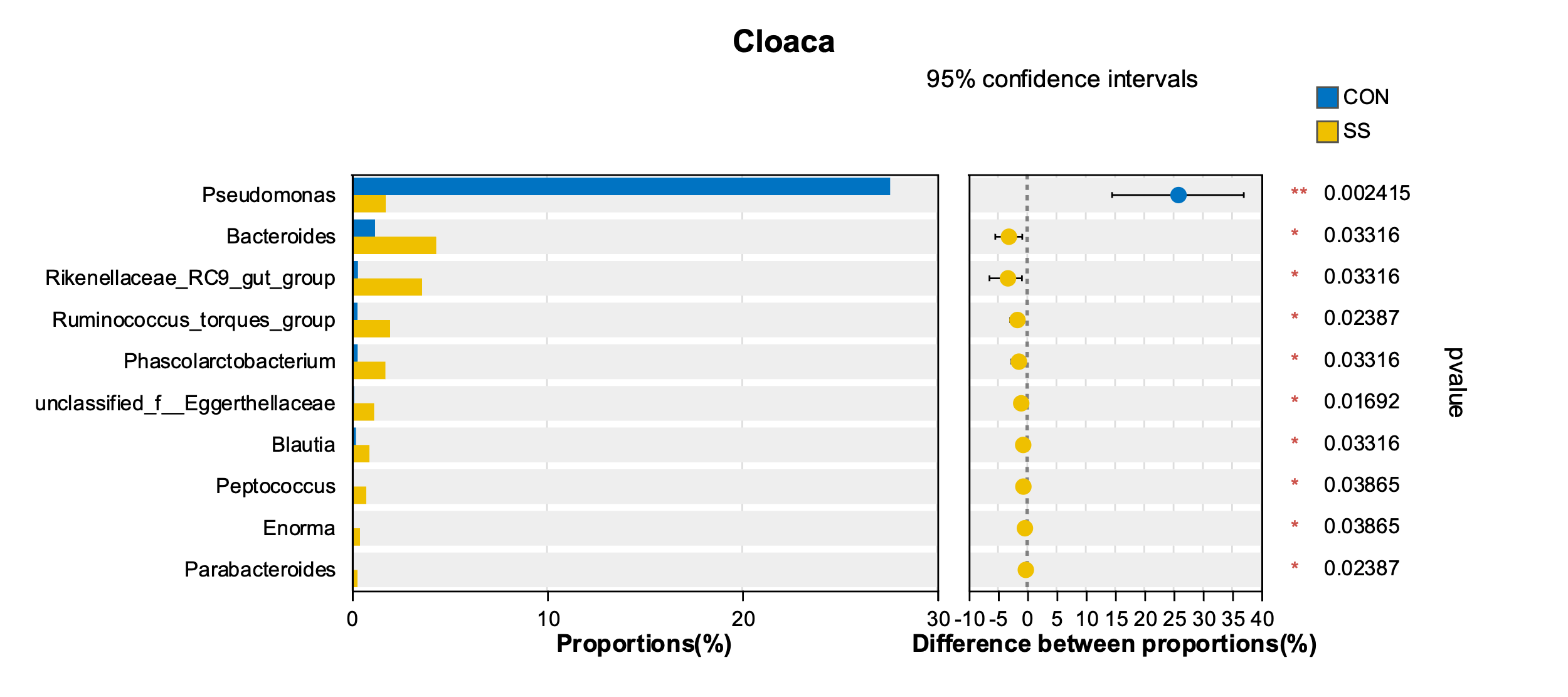


**Figure S1 Maternal microbes transfer to the eggshell and yolk sac.** (A) A source-tracking analysis was performed on the microbial communities of the eggshell, breeder hen intestine, magnum, and cloaca. The left panel shows the proportions contributed by the magnum (*n* = 15), cloaca (*n* = 16), and intestine (*n* = 16) to the eggshell microbiota (*n* = 16). The Venn diagram at the genus level illustrates the bacterial genera shared among the eggshell, breeder hen intestine, magnum, and cloaca. Panels a-d indicate the identities and relative proportions of these shared genera. (B) The Venn diagram at the genus level shows the bacterial genera shared among the eggshell, yolk sac, meconium, and breeder hen cloaca. Panels a and b highlight the identities and relative proportions of the shared genera. (C) Spearman correlation-based similarity analysis of multiple tissues in maternal and offspring.


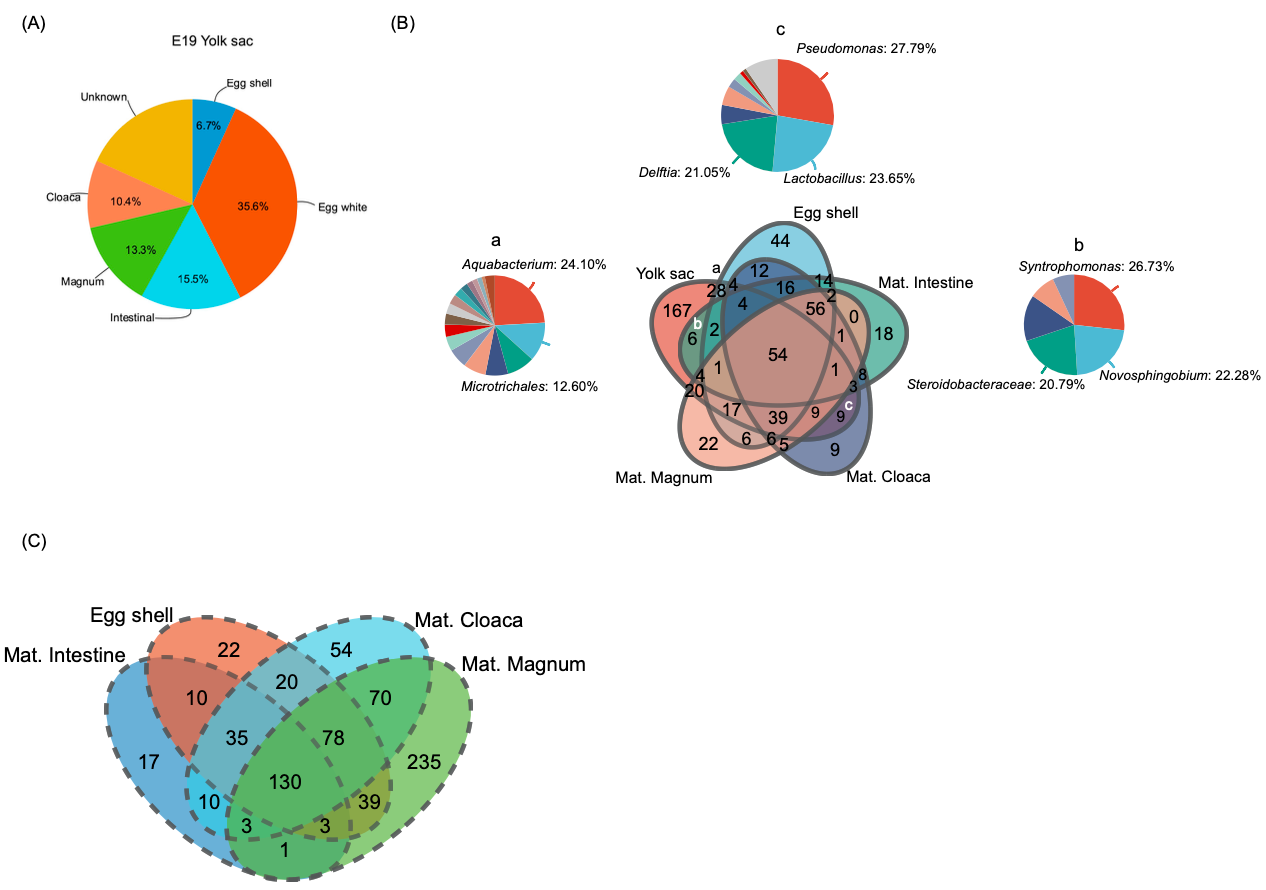


**Figure S2 The shared bacterial genera between maternal and offspring, as well as among different embryonic compartments.** (A) SourceTracker analysis indicates the microbial origins of the yolk sac microbiota. (B) Number and types of shared bacterial genera between the yolk sac (*n* = 15) and the egg shell (*n* = 16), magnum (*n* = 15), cloaca (*n* = 16), and intestine (*n* = 16). (C) Venn diagram illustrating the similarity in bacterial genera among the intestine, egg shell, cloaca, and magnum.


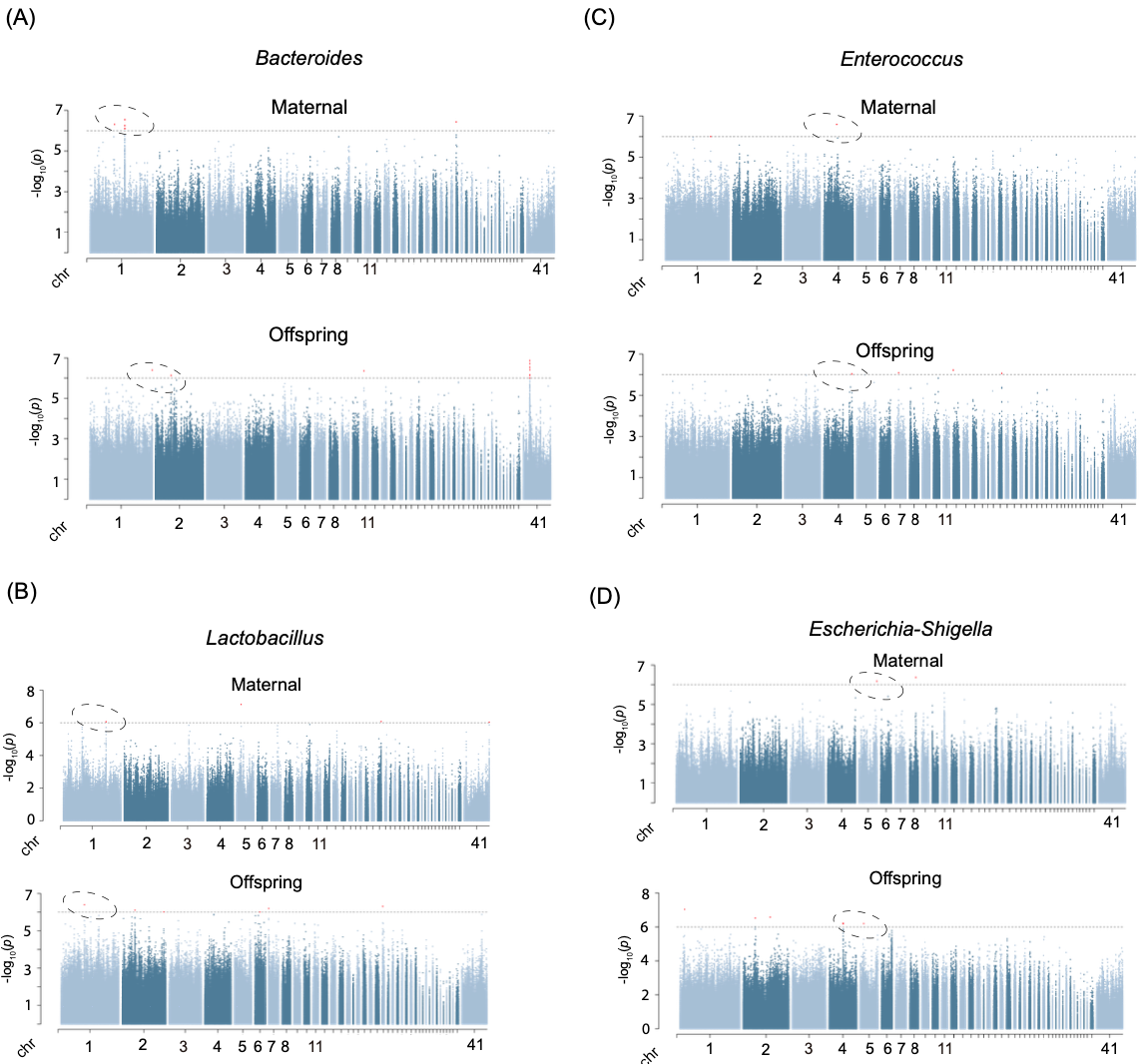
**Figure S3 The heritability and significant variants of the phenotype in maternal and offspring.** (A-D) Manhattan plot of *Bacteroides, Lactobacillus, Enterococcus, Escherichia-Shigella*. The significance threshold was 1/nSingle Nucleotide Polymorphism (SNP) = 1e-06.


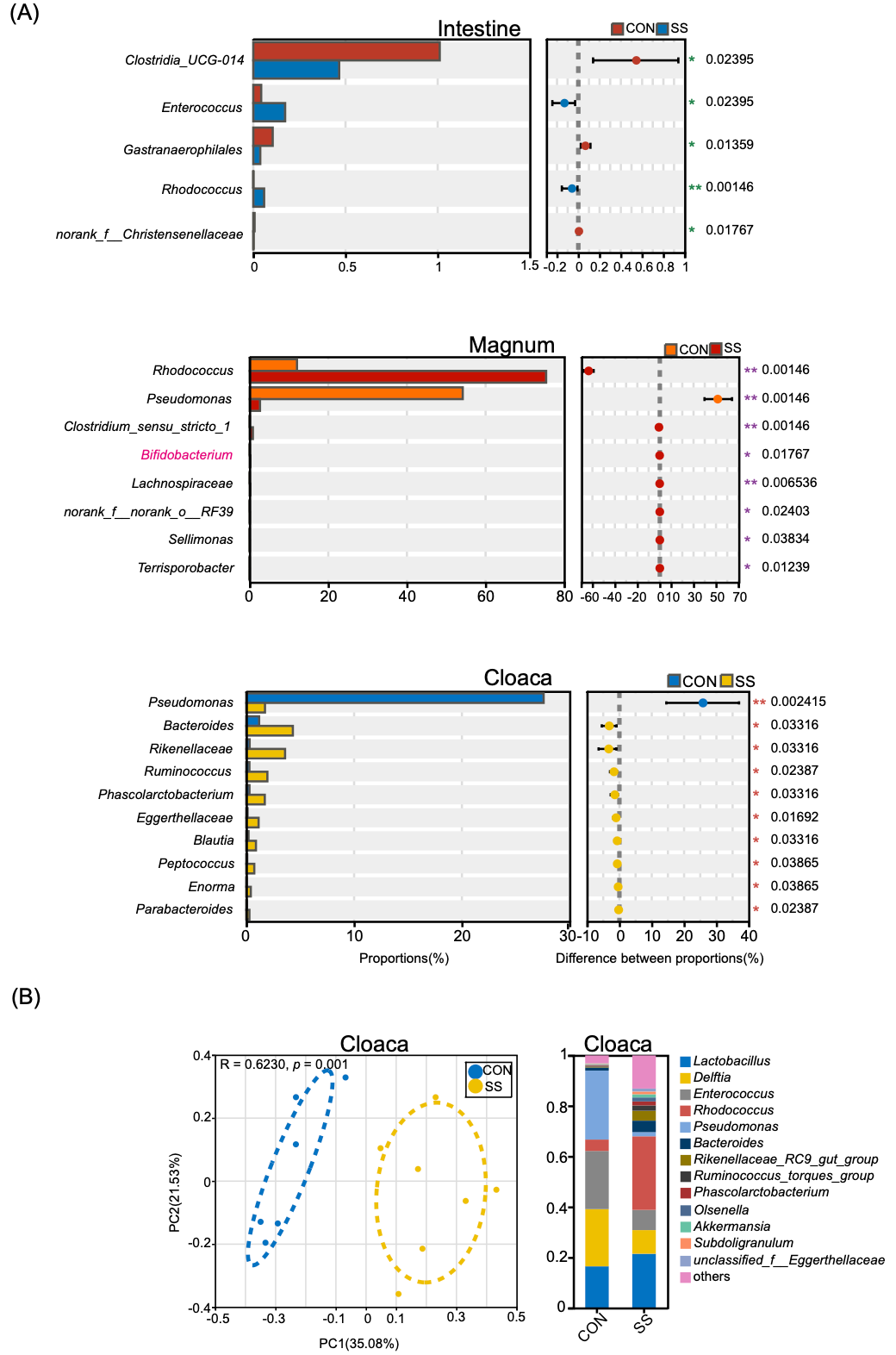


**Figure S4 Differential bacteria in breeder chicken's intestine, magnum, and cloaca.** (A) Bar charts depicting beta diversity and relative abundance in the cloaca. (B) Wilcoxon rank-sum tests applied to samples from the intestine, magnum, and cloaca.


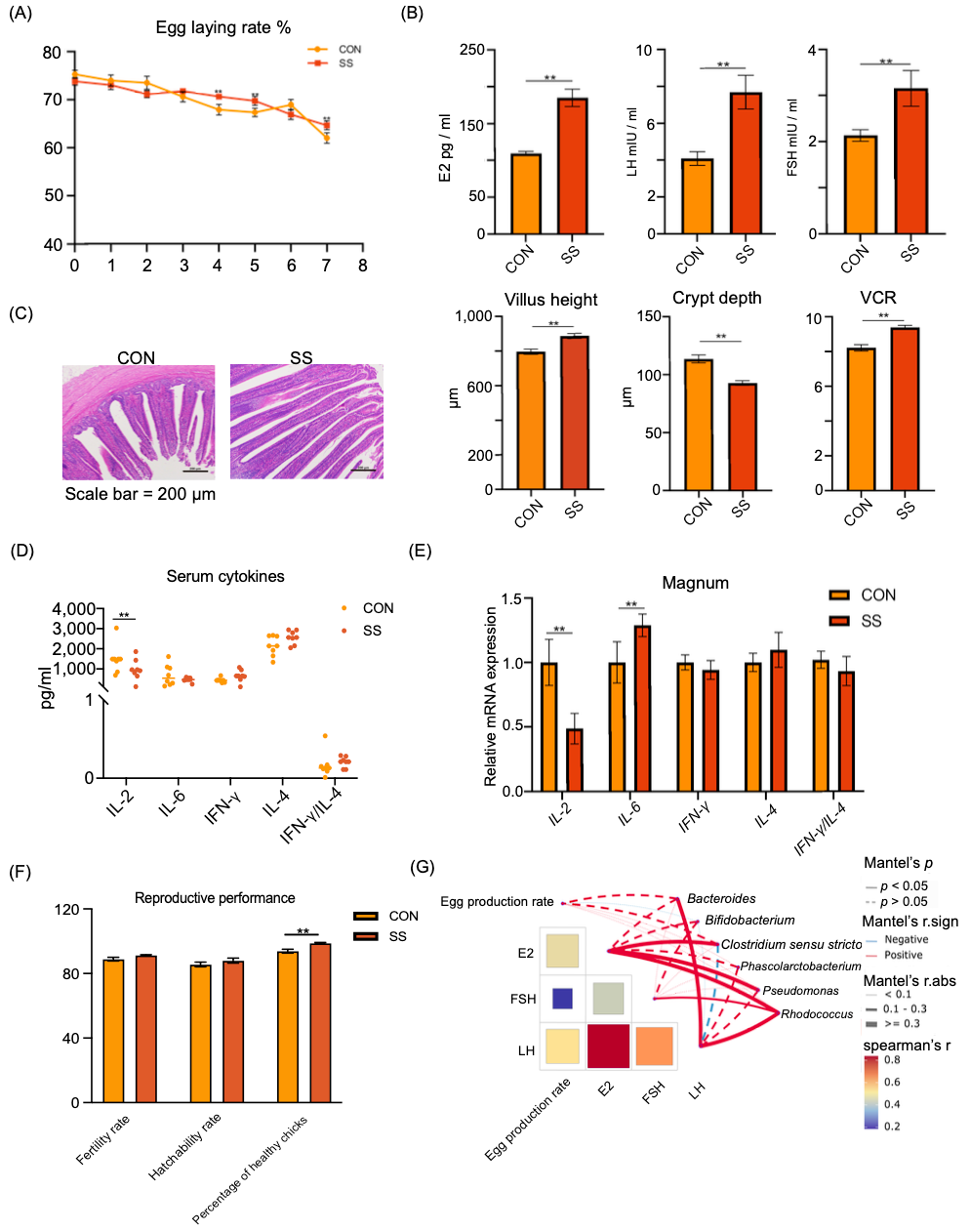
**Figure S5 Impact of dietary soyasaponin (SS) supplementation on the health of the intestinal and reproductive tracts in broiler breeders.** (A) Average egg production rate at 7 weeks (*n* = 8). Data are shown as mean ± SEMs. (B) Serum levels of Estradiol (E2), Luteinizing Hormone (LH), and Follicle-Stimulating Hormone (FSH) (*n* = 8). Data are shown as mean ± SEMs. (C) Morphology and statistical analysis of the ileum using H&E staining, bar = 200 μm. (D and E) Serum cytokine levels and mRNA expression of cytokines in the magnum (*n* = 8). Data are shown as mean ± SEMs. (F) Statistical analysis of reproductive performance parameters in breeder chickens. (G) Correlation analysis between average egg production rate, E2, LH, FSH, and various bacterial genera in the maternal reproductive tract, with color depth indicating correlation strength.


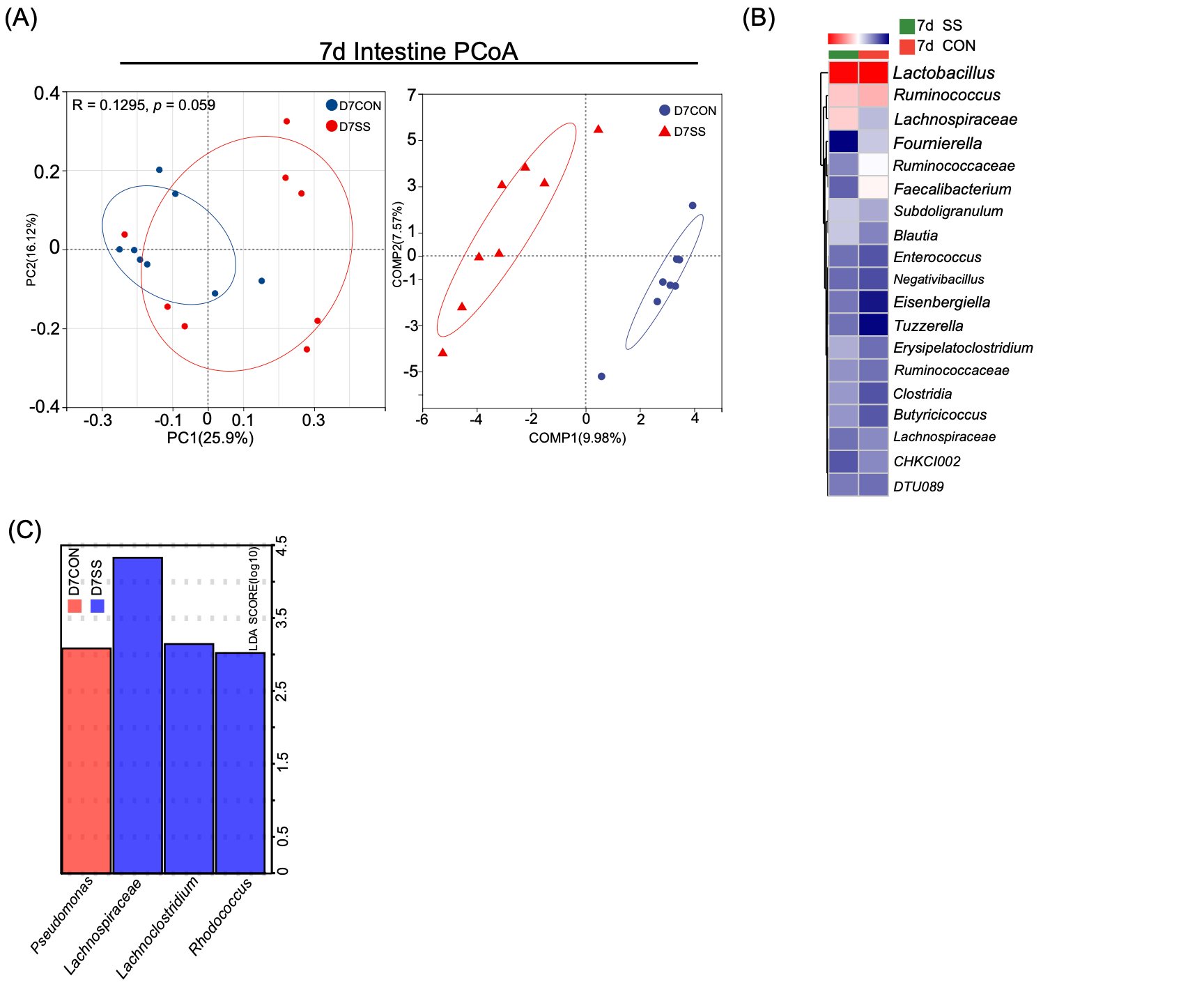


**Figure S6 Intestinal microbiota at 7 days in broiler chicken offspring.** (A) Principal coordinates analysis (PCoA) and Partial least squares discriminant analysis (PLS-DA) analysis results of the intestinal microbiota in 7-day-old broiler chicken offspring. (B and C) Heatmap at the genus level and differential bacterial genera in 7-day-old offspring.


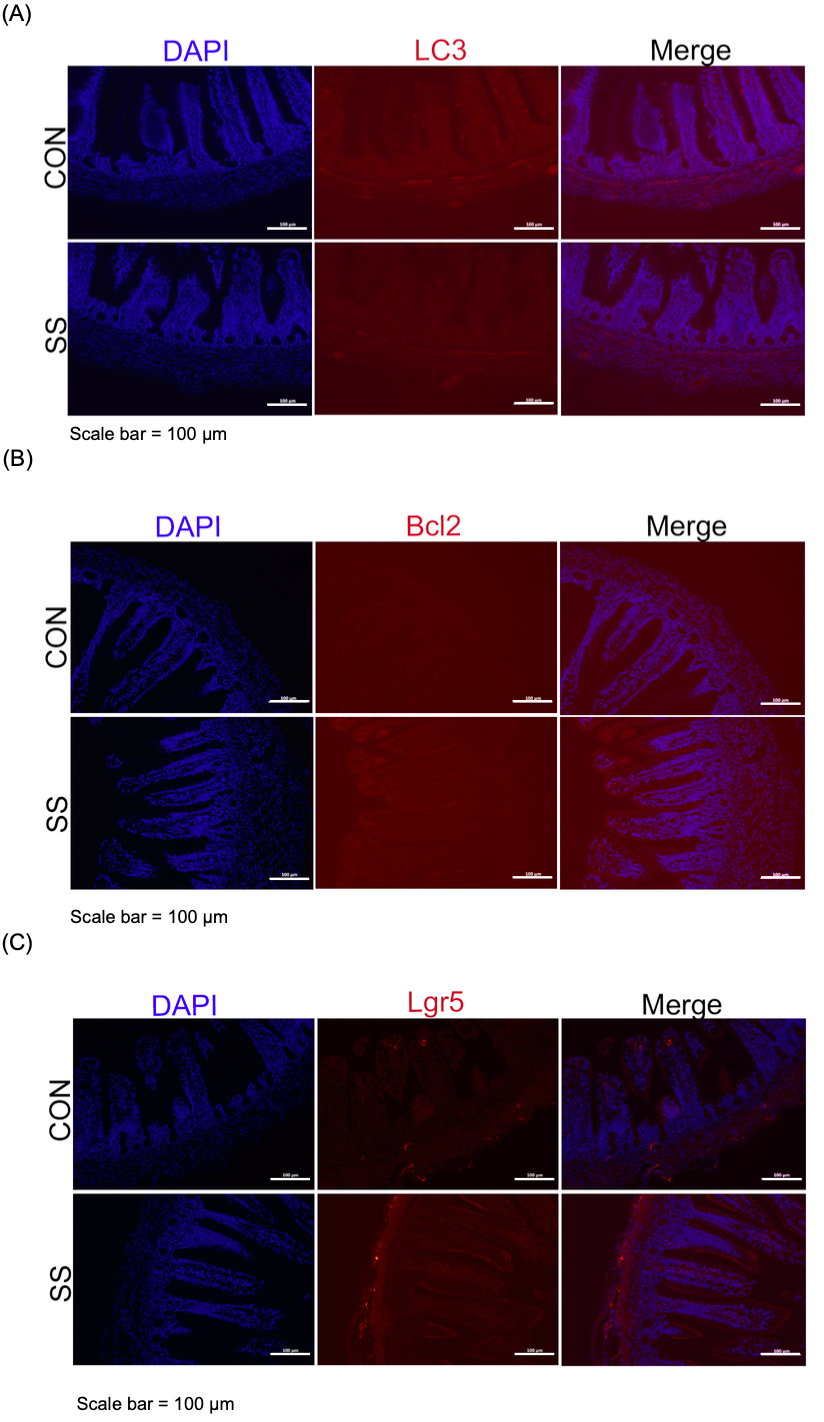


**Figure S7 D1 offspring intestinal immune fluorescence results.** (A) Immunofluorescence results of Microtubule-associated protein 1A/1B-light chain 3 (LC3). (B) B-cell lymphoma 2 (Bcl2). (C) Leucine-rich repeat-containing G-protein coupled receptor 5 (Lgr5) in the intestine of CON and SS groups, bar = 100 μm.


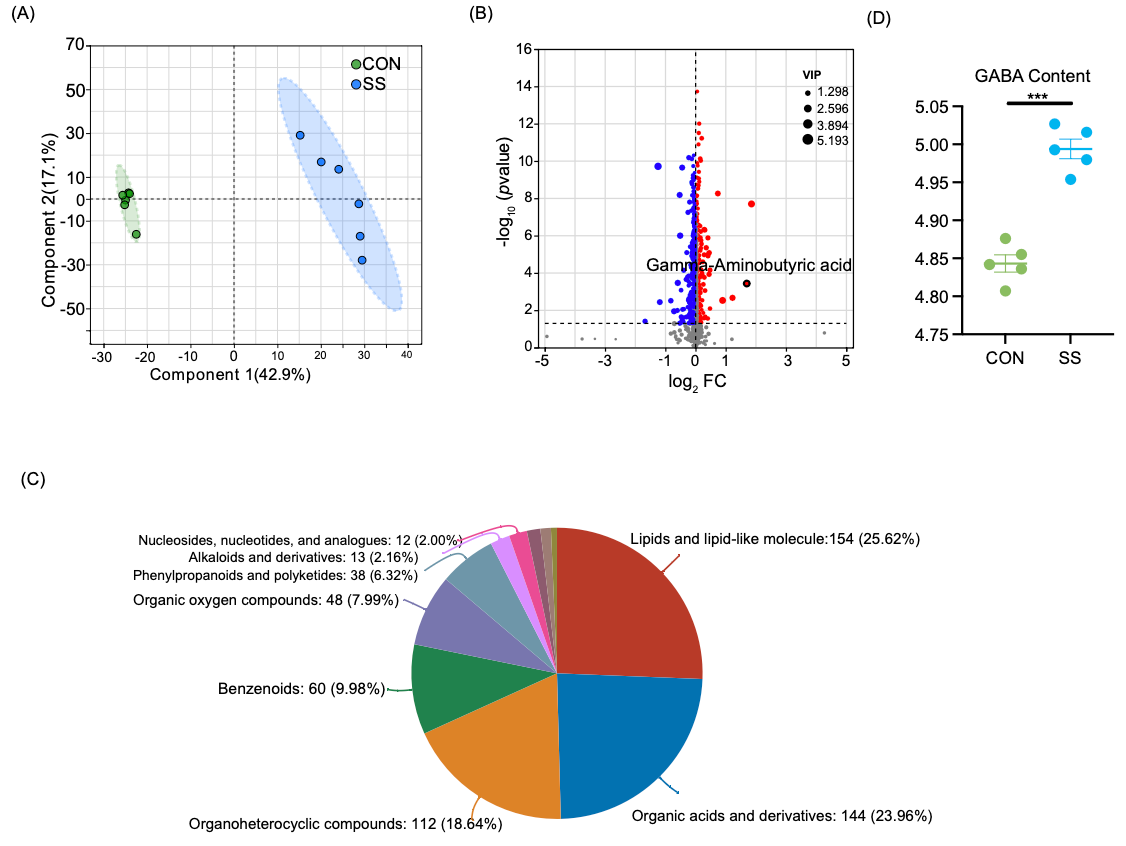


**Figure S8 Metabolomic structure of meconium.** (A) PLS-DA typification of the neonatal meconium metabolome. (B) Volcano plot of differential metabolites in meconium. (C) Annotation results for differential metabolites using the Human Metabolome Database (HMDB) database. (D) Comparative analysis of Gamma-aminobutyric acid (GABA) differences.


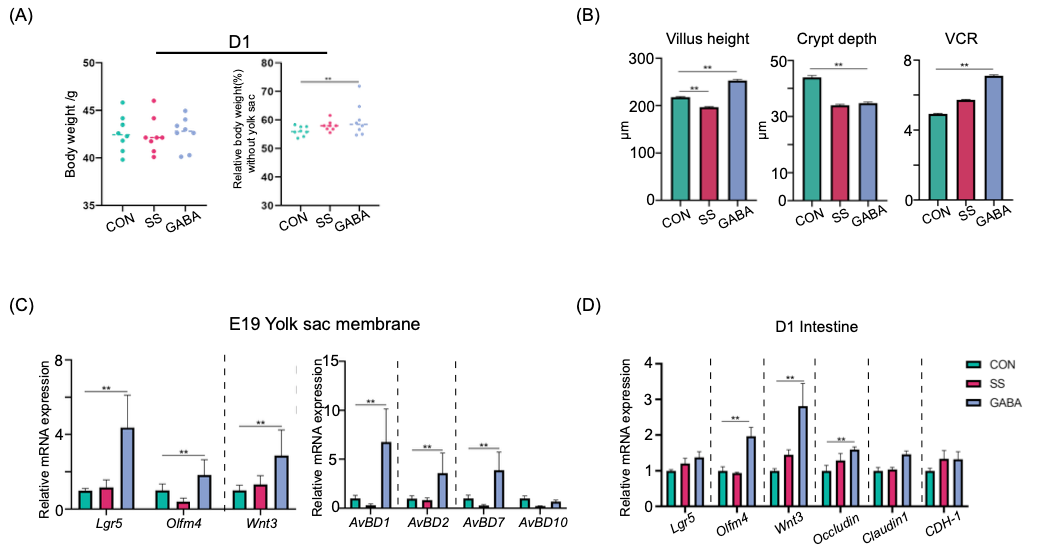


**Figure S9 In-ovo injection results.** (A) Average body weight, and relative body weight without the yolk sac of 1-day-old chicks (*n* = 8). Data are shown as mean ± SEMs. (B) Measurement of villus height (VH), crypt depth (cd), and villus-crypt ratio (VCR) on day 1 (*n* = 64). (C) Relative mRNA expression of genes related to proliferation, differentiation, and Avian beta-defensin 1 (AvBD) in the yolk sac membrane (*n* = 8). Data are shown as mean ± SEMs. (D) Relative mRNA expression of genes related to proliferation, differentiation, and intestinal physical barrier in the ileum on day 1.


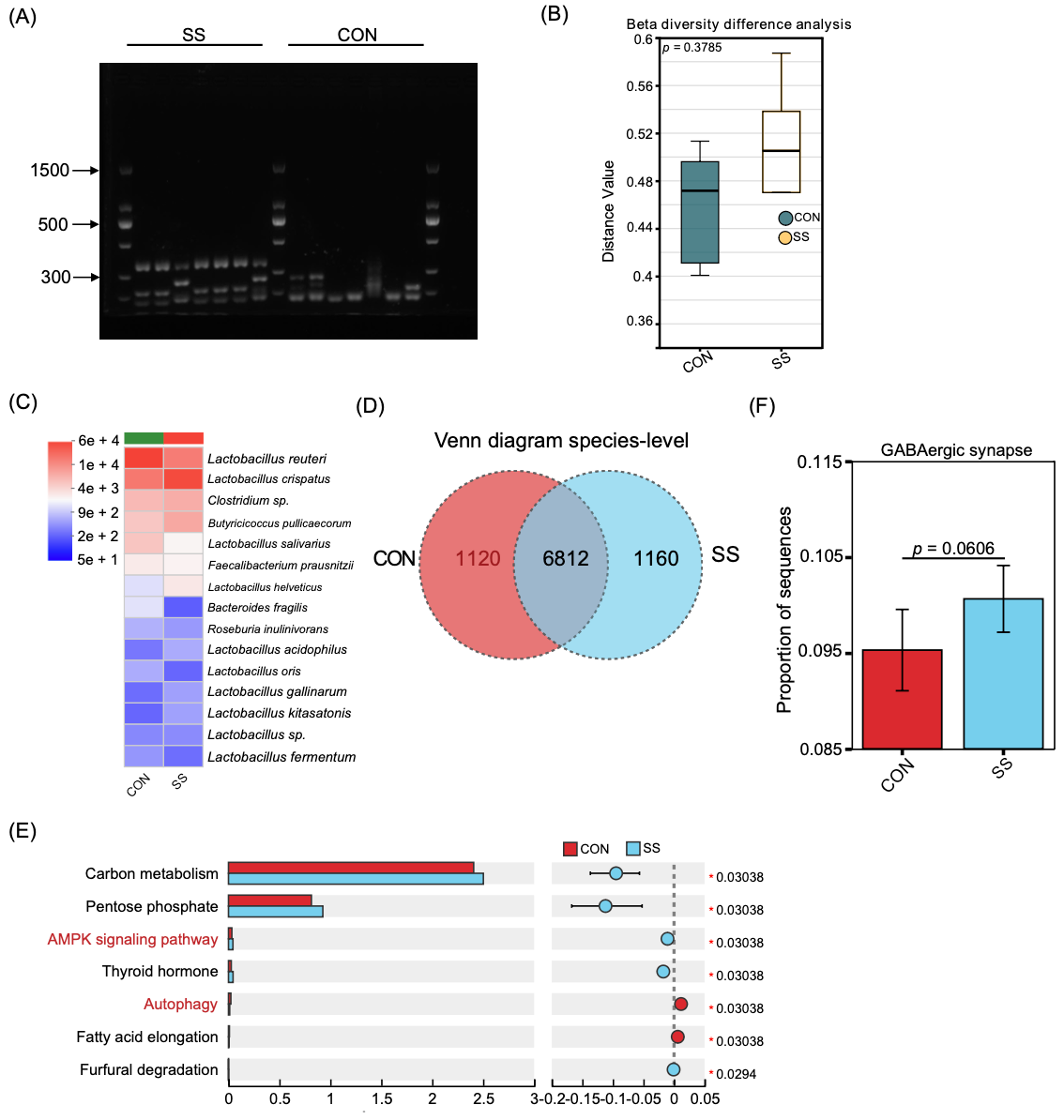


**Figure S10 *Bifidobacterium* levels from embryonic stage to 7 days post-hatch**. (A) Identification of the characteristic bacterial genus *Bifidobacterium* in the contents of the egg yolk sac by gel electrophoresis. (B) Beta diversity distances in the metagenomics of 7-day-old chicks. (C) Heatmap of species-level bacteria in 7-day-old chicks. (D) Venn analysis based on Transcripts per million (TPM) for the CON and SS groups. (E) Kyoto Encyclopedia of Genes and Genomes (KEGG) pathway differential analysis for 7-day-old chicks in the CON and SS groups. (F) Enrichment of the GABAergic synapse pathway in the metagenome.


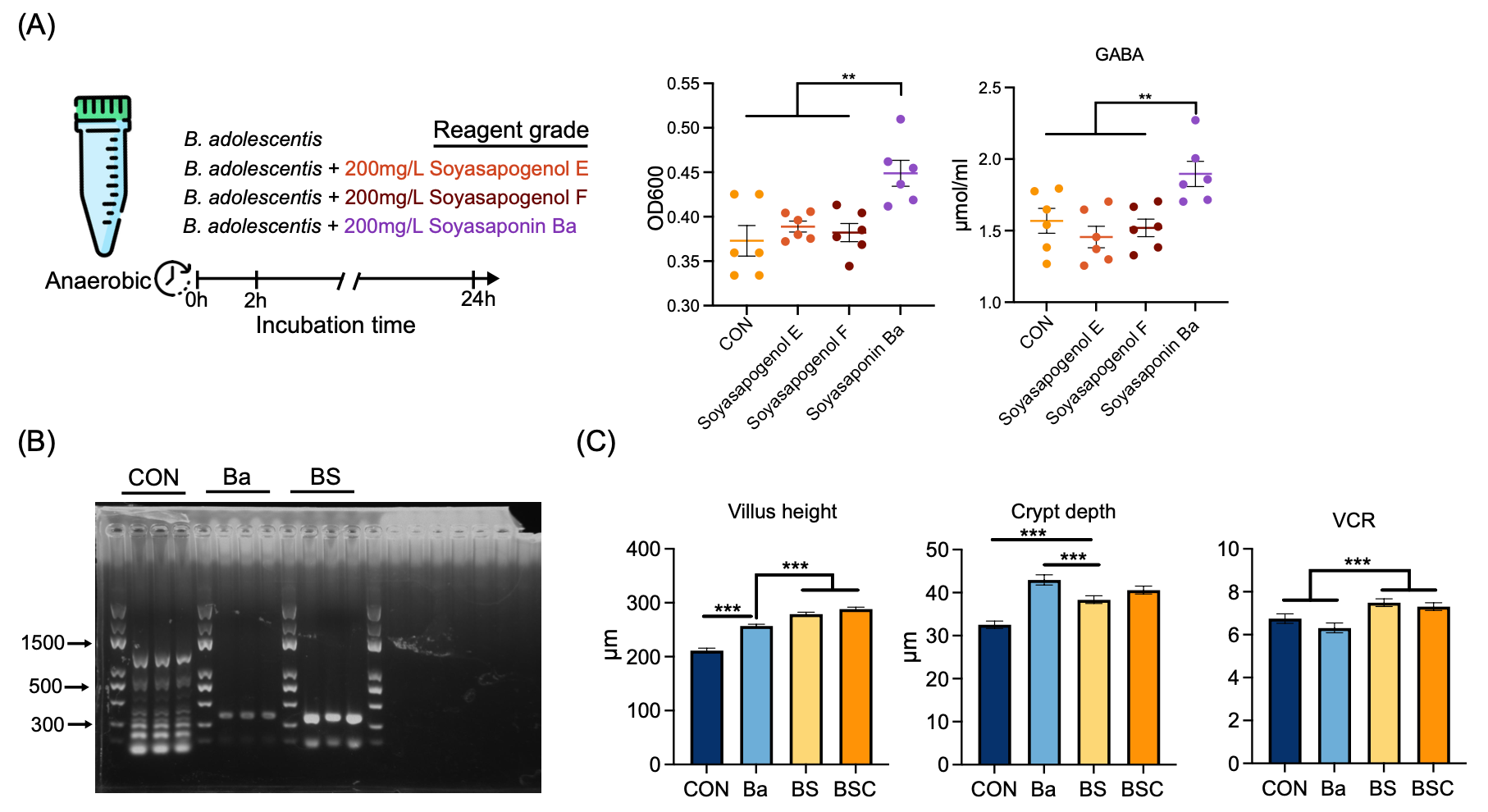


**Figure S11 In-ovo injection and co-culture results.** (A) Isolation of *Bifidobacterium adolescentis* and co-cultivation with feed grade SS. Co-culture results of reagent grade SS and its secondary metabolites (*n* = 6). Data are shown as mean ± SEMs. (B) PCR gel electrophoresis identification of Bifidobacterium-specific DNA in fecal samples. (C) VH, CD, and VCR statistics of intestinal villi morphology.


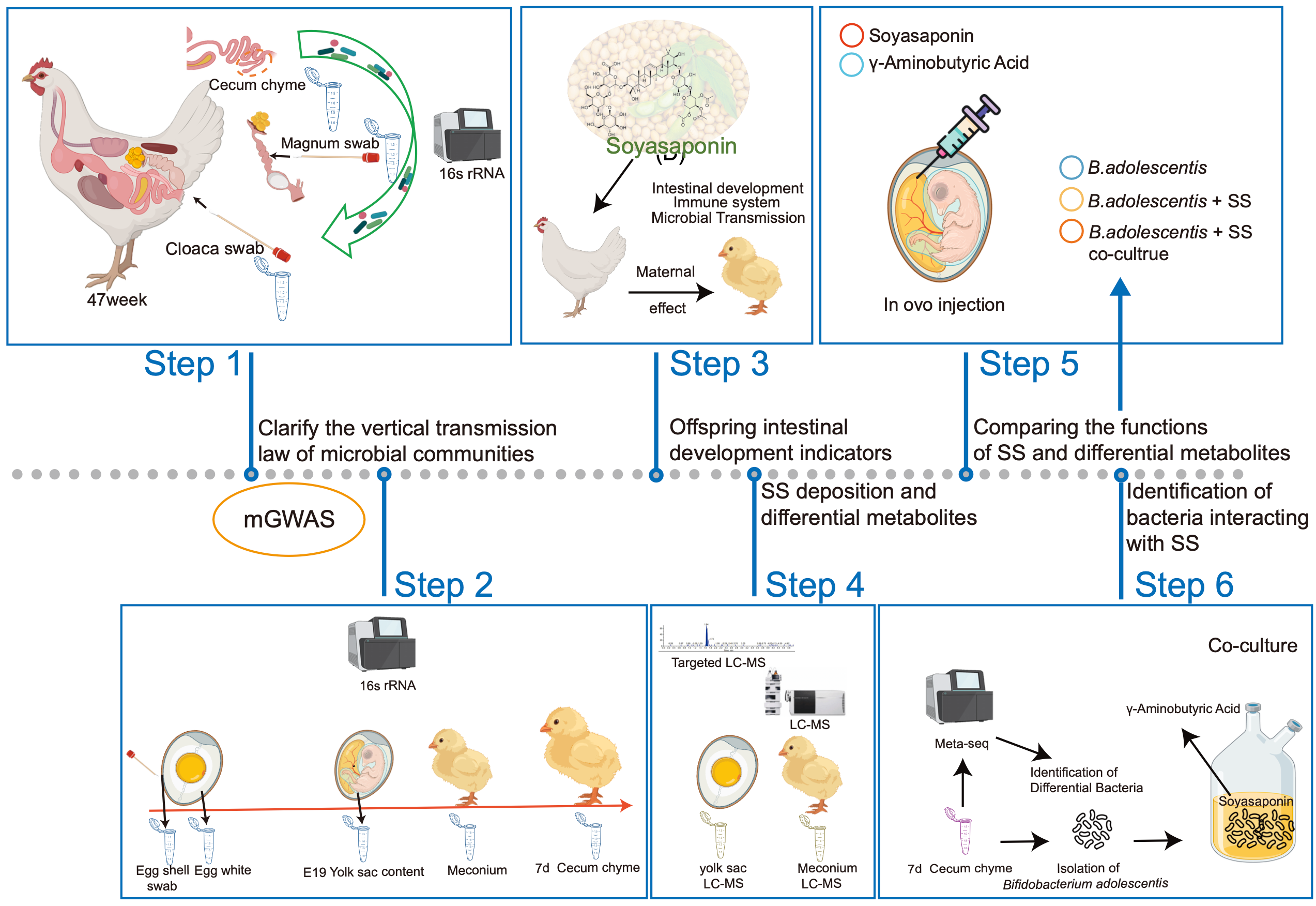


**Figure S12** **Flow diagram.** First, potential transmissible microbiota were identified by analyzing the intestinal tract, enlarged sac, and cloaca of breeder hens, in combination with the eggshell, egg white, yolk, meconium, and the gut microbiota of 7-day-old offspring. Then, microbiota genome-wide association studies (mGWAS) were performed to identify genetic variants associated with the transmissible microbiota. Next, dietary intervention with soybean saponins was conducted to explore its effects on the transmissible microbiota. Targeted yolk SS (soybean saponin) metabolomics and meconium metabolomics were used to identify differential metabolites. Chicken embryo injection experiments were then used to compare the effects of deposited SS and differential metabolites. Finally, the transmissible probiotics and their functional effects were clarified through culturomics combined with metagenomics.
